# A Novel *Toxoplasma* Inner Membrane Complex Suture-Associated Protein Regulates Suture Protein Targeting and Colocalizes with Membrane Trafficking Machinery

**DOI:** 10.1128/mBio.02455-21

**Published:** 2021-10-12

**Authors:** Jessica H. Chern, Rebecca R. Pasquarelli, Andy S. Moon, Allan L. Chen, Jihui Sha, James A. Wohlschlegel, Peter J. Bradley

**Affiliations:** a Department of Microbiology, Immunology and Molecular Genetics, University of California, Los Angeles, Los Angeles, California, USA; b Molecular Biology Institute, University of California, Los Angeles, Los Angeles, California, USA; c Department of Biological Chemistry and Institute of Genomics and Proteomics, David Geffen School of Medicine, University of California, Los Angeles, Los Angeles, California, USA; University of Arizona

**Keywords:** Apicomplexa, BioID, inner membrane complex, *Toxoplasma gondii*, dynamin-related protein

## Abstract

The cytoskeleton of Toxoplasma gondii is composed of the inner membrane complex (IMC) and an array of underlying microtubules that provide support at the periphery of the parasite. Specific subregions of the IMC carry out distinct roles in replication, motility, and host cell invasion. Building on our previous *in vivo* biotinylation (BioID) experiments of the IMC, we identified here a novel protein that localizes to discrete puncta that are embedded in the parasite’s cytoskeleton along the IMC sutures. Gene knockout analysis showed that loss of the protein results in defects in cytoskeletal suture protein targeting, cytoskeletal integrity, parasite morphology, and host cell invasion. We then used deletion analyses to identify a domain in the N terminus of the protein that is critical for both localization and function. Finally, we used the protein as bait for *in vivo* biotinylation, which identified several other proteins that colocalize in similar spot-like patterns. These putative interactors include several proteins that are implicated in membrane trafficking and are also associated with the cytoskeleton. Together, these data reveal an unexpected link between the IMC sutures and membrane trafficking elements of the parasite and suggest that the suture puncta are likely a portal for trafficking cargo across the IMC.

## INTRODUCTION

The phylum Apicomplexa is composed of obligate intracellular parasites that cause substantial disease in humans and animals worldwide (1). The most prominent apicomplexans that infect humans are Toxoplasma gondii, which causes disease in immunocompromised individuals and neonates, *Plasmodium* spp., the causative agents of malaria, and *Cryptosporidium* spp., which cause diarrheal diseases in children (2–4). Important animal pathogens include Neospora caninum, which causes abortion in cattle and neurological disease in dogs, and *Eimeria* spp., which cause disease in poultry (5). These parasites share a number of unique organelles that enable them to infect and replicate within their mammalian host cells (6). Because these organelles and many of their constituents are unique to the pathogens, they make attractive targets for the development of therapeutics that can specifically target the parasite.

One of these organelles is the inner membrane complex (IMC), which lies beneath the parasite’s plasma membrane and consists of both membrane and cytoskeletal elements (7). The IMC is additionally supported by a series of microtubules that are tethered to the basket-shaped conoid at the apical end of the parasite and extend nearly the length of the cell. The IMC is known to carry out three major functions in infection of host cells and intracellular replication. First, it hosts the glideosome, an actin-myosin motor that interacts with adhesins secreted onto the parasite’s surface for motility and invasion (8). Second, it serves as a scaffold for the formation of daughter cells via the internal budding process known as endodyogeny (6). Finally, the apical cap portion of the organelle has recently been shown to support the conoid, a microtubule-based structure at the extreme apex of the cell which controls the release of secretory proteins for host cell invasion (9, 10). While these important activities of the IMC have been described and some of the key players identified, the precise roles of many of the constituents of the IMC have yet to be determined.

The IMC is able to carry out its diverse functions by partitioning the organelle into distinct subcompartments, each containing its own cargo of proteins (6, 7). The glideosome components that power motility are localized to the membrane vesicles of the IMC body and apical cap, with the motor facing the plasma membrane to tether to secreted micronemal adhesins (8). The apical cap portion of the organelle hosts the AC9/AC10/ERK7 complex, which regulates the stability of the conoid, which in turn is essential for release of the micronemes and rhoptries for attachment and penetration, respectively (9, 10). During endodyogeny, distinct groups of IMC proteins are synthesized in a “just-in-time” approach in which proteins are sequentially synthesized and added onto the membranes and cytoskeleton of forming daughter buds for the replication of new cells (11). The membrane vesicles of the IMC body are arranged into rectangular plates that are stitched together by the IMC suture proteins, which appear to be important for maintaining parasite shape and ensuring faithful replication (12, 13). IMC suture proteins are also associated with the parasite’s cytoskeletal network, although how they are arranged into their rectangular pattern and are tethered to the cytoskeleton is unknown. The basal complex is at the extreme base of the IMC and plays key roles in the expansion of the forming daughter buds and constriction of the daughters during the final stages of division (14). Lastly, the apical annuli are a series of five discrete spots anchored to the cytoskeleton between the apical cap and the parasite body, which may serve as pores for the transfer of nutrients or the removal of waste across the IMC (15).

One important advance in the discovery of many of the IMC proteins is the use of *in vivo* biotinylation (BioID) with bait proteins targeted to the various subcompartments, which can be used to identify new proximal and interacting proteins labeled in each location (12, 15–17). In this study, we characterize a protein identified in our previous IMC BioID experiments that surprisingly localizes to a series of puncta that colocalize with the IMC sutures and associate with the cytoskeleton (12, 13). Gene knockout of the protein showed that it is important for trafficking of a subgroup of the cytoskeletal suture proteins and integrity of the cytoskeleton, which results in defects in parasite morphology, replication, and host cell invasion. Deletion analyses demonstrated that the coiled-coil domains of the protein are surprisingly dispensable, but a conserved N-terminal region is essential for localization and function. We then used the protein as bait for BioID experiments, which identified several other proteins that colocalize with these puncta and are associated with the cytoskeleton. Among these are proteins that are implicated in membrane transport, suggesting that the IMC suture puncta tether vesicle trafficking machinery for delivery of cargo into or across the IMC.

## RESULTS

### TGGT1_202220 localizes to distinct cytoskeletal puncta along the IMC sutures.

**FIG 1:**
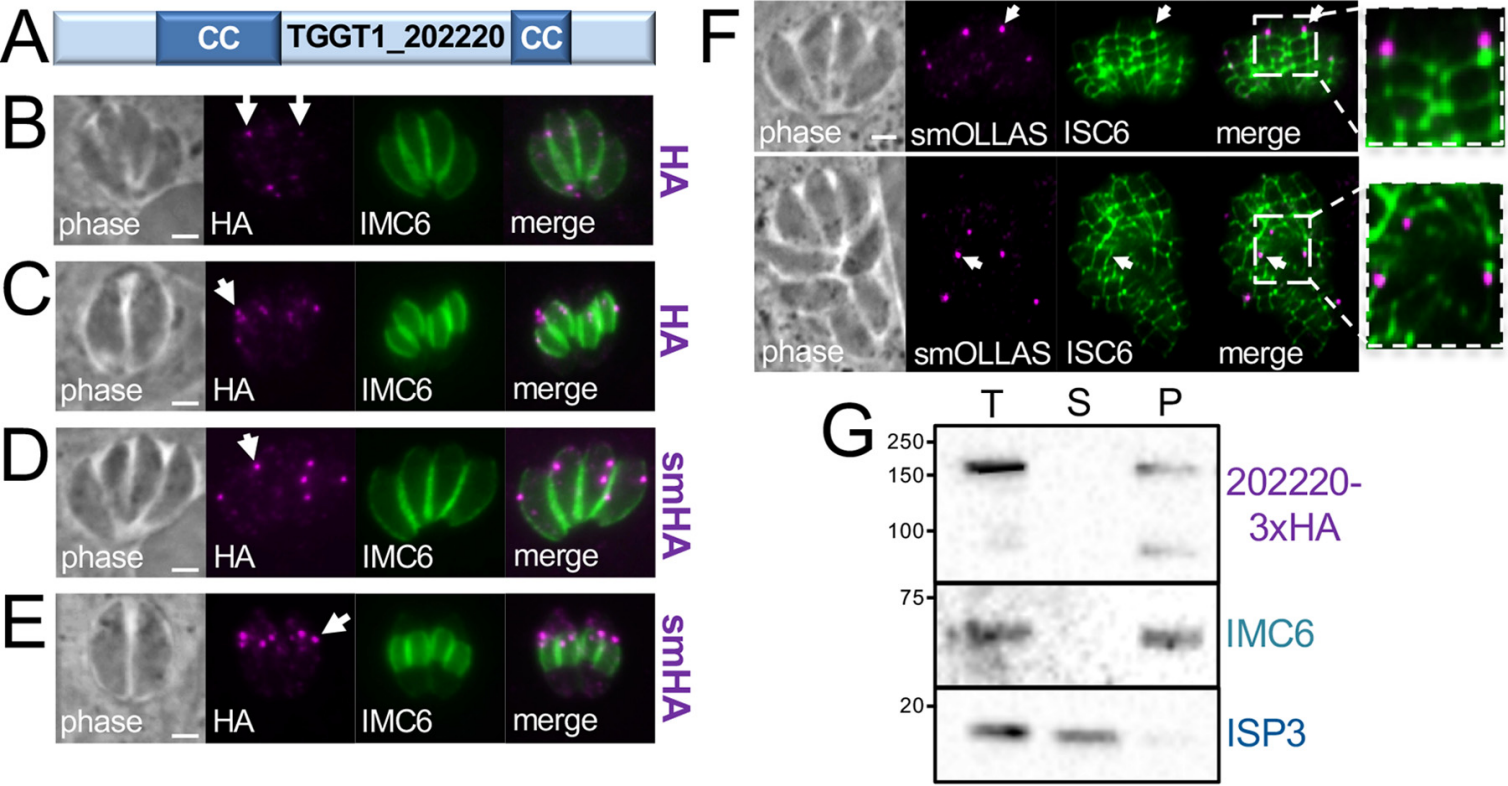
TGGT1_202220 localizes to cytoplasmic spots along the IMC sutures and is associated with the cytoskeleton. (A) Diagram of TGGT1_202220 showing two predicted coiled-coil (CC) domains. (B) IFA of endogenously 3×HA-tagged TGGT1_202220 showing several faint puncta in the cytoplasm (arrows). Magenta, mouse anti-HA; green, rabbit anti-IMC6. (C) IFA showing that the cytoplasmic puncta appear near the apical ends of the developing daughter buds during endodyogeny (arrow). Magenta, mouse anti-HA; green: rabbit anti-IMC6. (D) IFA showing endogenous tagging of TGGT1_202220 with spaghetti monster HA (smHA) enables better detection of the cytoplasmic puncta (arrow). Magenta, mouse anti-HA; green, rabbit anti-IMC6. (E) TGGT1_202220 tagged with smHA also shows enhanced staining near the developing daughter buds (arrow). Magenta, mouse anti-HA; green, rabbit anti-IMC6. (F) IFA showing that smOLLAS-tagged TGGT1_202220 puncta colocalize with the IMC sutures (ISC6-3×HA). The arrow in the top panel shows a punctum on a longitudinal suture. The arrow in the bottom panel points to a punctum on a transverse suture. Insets shows magnifications of the boxed regions highlighting suture colocalization. Magenta, rat anti-OLLAS; green, mouse anti-HA. (G) Western blot analysis of TX-100 detergent fractionation shows that 3×HA-tagged TGGT1_202220 partitions to the cytoskeletal pellet with the alveolin IMC6 and is not released like the membrane-associated IMC protein ISP3. T, total; S, detergent-soluble supernatant; P, detergent-insoluble cytoskeletal pellet. Bars = 2 μm.

Our previous BioID experiments using IMC proteins as bait identified a large number of candidate IMC proteins, many of which have been verified by epitope tagging (9, 12, 13). One hit that was identified in BioID experiments using both AC9 and ISP3 proteins as bait was TGGT1_202220. TGGT1_202220 has a predicted mass of 122 kDa and lacks identifiable protein domains other than two internal coiled-coil (CC) domains (Fig. 1A) (18). TGGT1_202220 is restricted to T. gondii and its closest relatives, as orthologues are present in Hammondia hammondi, Neospora caninum, and Besnoitia besnoiti but not in *Eimeria* spp. or *Sarcocystis* spp. (see Fig. S1 in the supplemental material) (19). The regions of highest similarity appeared to be within the CC domains as well as in the N- and C-terminal regions of the protein outside the CC domains. To determine the localization of TGGT1_202220, we endogenously tagged the protein with a three-hemagglutinin (3×HA) tag which showed a series of faint spots in the parasite’s cytoplasm, some of which were close to background staining (Fig. 1B). In parasites that were dividing by endodyogeny, the spots appeared brighter, in agreement with cell cycle expression data (Fig. 1C) (11). To better assess localization of the protein, we used a spaghetti monster HA epitope tag (smHA), which enhances detection via its 10 HA tags buried in a nonfluorescent green fluorescent protein (GFP) backbone (20). This revealed a substantially enhanced signal of the cytoplasmic spots (Fig. 1D), which again were brighter in dividing parasites (Fig. 1E).

10.1128/mBio.02455-21.1 FIG S1 Alignment of TGGT1_202220 orthologues. Clustal Omega alignment of TGGT1_202220 orthologues from *H. hammondi*, N. caninum, and *B. besnoiti* (19, 52). An asterisk indicates a fully conserved residue, whereas a colon indicates strong conservation and a period indicates weaker similarity. The positions of the coiled-coil domains are noted above the alignment with thick blue lines. Download FIG S1, TIF file, 1.8 MB. Copyright © 2021 Chern et al. 2021 Chern et al. https://creativecommons.org/licenses/by/4.0/ This content is distributed under the terms of the Creative Commons Attribution 4.0 International license.

Since the spotted localization of TGGT1_202220 differs from the peripheral localization of most IMC proteins, we examined structures associated with the IMC, including the cortical microtubules, apical annuli, and IMC sutures using smOLLAS (spaghetti monster Escherichia coli OmpF linker and mouse langerin fusion sequence)-tagged TGGT1_202220 (21). While we did not observe colocalization of the puncta with the microtubules or apical annuli (Fig. S2), the spots did colocalize with the IMC sutures (Fig. 1F). The signal was most frequently detected on the longitudinal sutures just adjacent to the intersection of the longitudinal and transverse sutures, but it was sometimes seen on the transverse sutures as well. We then used detergent fractionation to determine if the protein is tethered to the parasite’s cytoskeleton, using IMC6 and ISP3 as controls for the cytoskeletal and membrane fractions, respectively (Fig. 1G) (12). While the protein reproducibly suffered from some breakdown during fractionation, the signal clearly partitioned with the insoluble cytoskeleton and was not released by detergent extraction. Together, these results indicate that TGGT1_202220 associates with the cytoskeleton at the IMC sutures, and we thus named the protein IMC suture-associated protein 1 (ISAP1).

10.1128/mBio.02455-21.2 FIG S2 TGGT1_202220 does not colocalize with the subpellicular microtubules or apical annuli. (A) IFA showing that TGGT1_202220-smOLLAS does not colocalize with the subpellicular microtubules seen by 3×HA tagging of TGGT1_248740, which localizes to the subpellicular microtubules beneath the apical cap (13). Magenta, rat anti-OLLAS; green, rabbit anti-HA. (B) IFA showing that TGGT1_202220-smOLLAS does not colocalize with the apical annuli observed via AAP3 3×HA-tagging (15). Magenta, rat anti-OLLAS; green, rabbit anti-HA. Bars = 2 μm. Download FIG S2, TIF file, 0.8 MB. Copyright © 2021 Chern et al. 2021 Chern et al. https://creativecommons.org/licenses/by/4.0/ This content is distributed under the terms of the Creative Commons Attribution 4.0 International license.

### Disruption of ISAP1 substantially affects the parasite’s lytic cycle.

**FIG 2:**
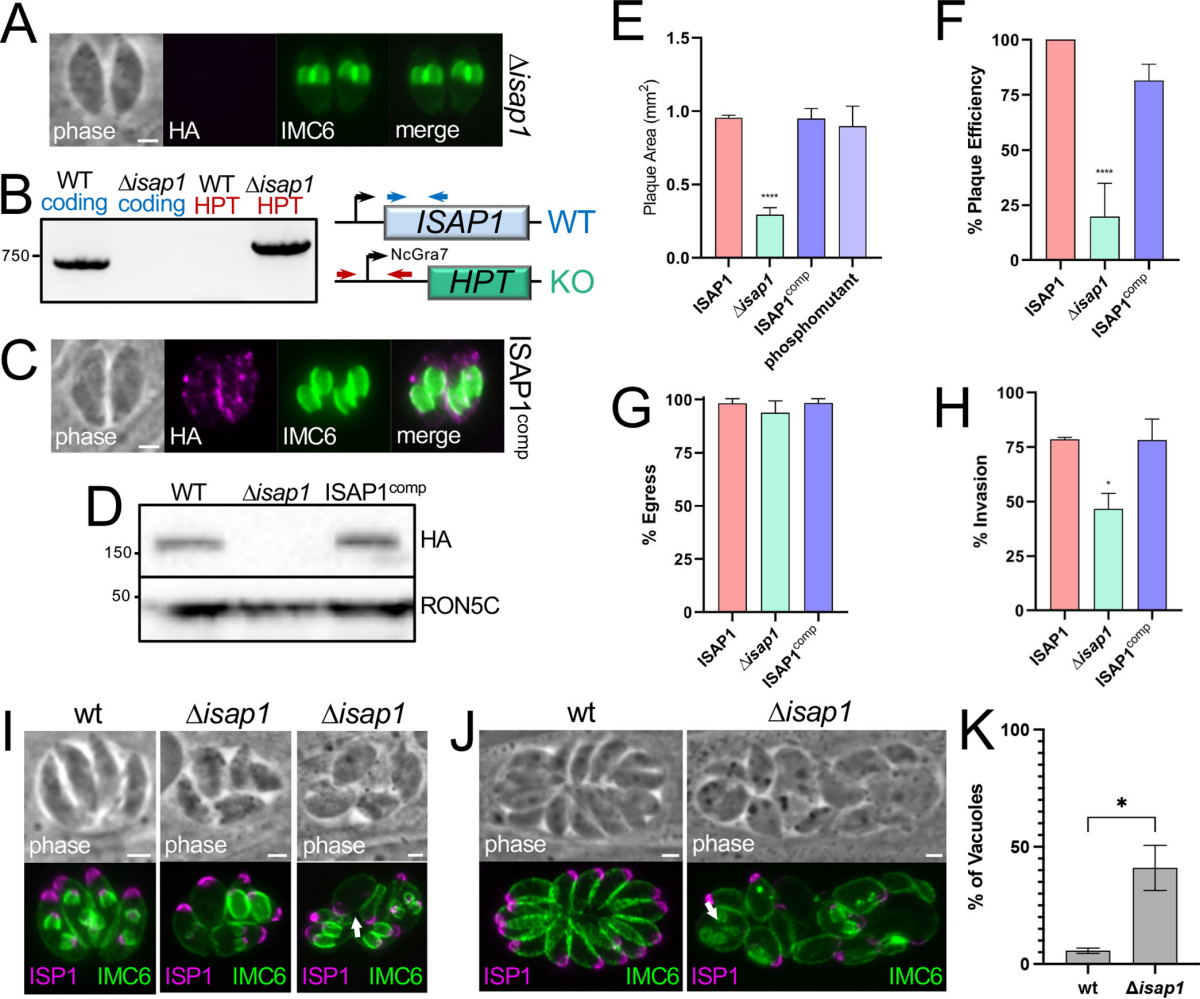
Gene knockout of ISAP1. (A) IFA showing lack of HA staining in Δ*isap1* parasites. Magenta, mouse anti-HA; green, rabbit anti-IMC6. (B) PCR and diagram showing that the Δ*isap1* strain contains the correct amplicon for the replacement of ISAP1 with the selectable marker hypoxanthine-xanthine-guanine phosphoribosyl transferase (HPT) and lacks the ISAP1-coding amplicon. Primer positions are shown with arrows, and amplicons agree with the anticipated sizes for the knockout using wild-type genomic DNA as a control. (C) Complementation with the ISAP1 coding sequence driven from the ISC6 promoter restores the spot-like pattern similar to the wild-type protein. Magenta, mouse anti-HA; green, rabbit anti-IMC6. (D) Western blot analysis of HA-tagged Δ*isap1* and complemented strains shows that complementation restores levels of the ISAP1 protein similar to that seen for the HA-tagged strain. (E) Quantification of plaque assays showing disruption of ISAP1 results in a 69% decrease in plaque size (****, *P < *0.0001). The defect is rescued by complementation (ISAP1^comp^). The ISAP1 phosphomutant (Fig. S3) also rescues the plaque defect. (F) Graph showing an 80% reduction in plaque efficiency of Δ*isap1* parasites (****, *P < *0.0001), which is mostly rescued upon complementation. (G) Ionophore induced egress is not significantly affected in Δ*isap1* parasites. (H) Host cell invasion is reduced by 42% in Δ*isap1* parasites (*, *P > *0.05). (I) IFA at 24 h postinfection showing that loss of ISAP1 results in swollen parasites that have dysregulated endodyogeny and morphological defects. The arrow points to a swollen parasite. Magenta, mouse anti-ISP1; green, rabbit anti-IMC6. (J) IFA at 32 h postinfection showing more severe defects in morphology and daughter cell formation. The arrow points to four daughter buds in a maternal parasite. Magenta, mouse anti-ISP1; green, rabbit anti-IMC6. (K) Quantification of vacuoles with misshapen parasites and/or dysregulated endodyogeny in Δ*isap1* parasites (*, *P < *0.05). Bars = 2 μm.

ISAP1 was assigned a phenotype score of −3.49 in the *Toxoplasma* genome-wide CRISPR/Cas9 screen (22), suggesting that the protein is either important for fitness or essential. To directly assess ISAP1 function, we used CRISPR/Cas9 to disrupt its gene from the HA epitope-tagged strain (23). Immunofluorescence assay (IFA) analysis of a clonal isolate of the knockout showed a loss of the HA staining, and the knockout was confirmed by PCR (Fig. 2A and B). We then complemented the Δ*isap1* parasites with the full-length ISAP1 gene driven from the ISC6 promoter, which restored the spot-like pattern in IFA and resulted in levels of expression similar to those of the wild-type tagged strain (Fig. 2C and D) (13). These results demonstrated that ISAP1 can be disrupted and that its staining pattern and protein levels can be restored by complementation.

10.1128/mBio.02455-21.3 FIG S3 Mutagenesis of the ISAP1 phosphorylation sites. (A) Diagram showing the position of ISAP1 phosphorylation sites (yellow bars, residues S74, T76, T149, S280, S856, S936, and S941) identified by phosphoproteomics. All 7 serine or threonine residues were mutated to alanine (19, 53). (B) IFA showing that the ISAP1 phosphomutant localizes to cytoplasmic puncta similarly to the wild-type complement protein. Rescue of the Δ*isap1* plaque defect by the phosphomutant is shown in Fig. 2E. Magenta, mouse anti-HA; green, rabbit anti-IMC6. Bars = 2 μm. Download FIG S3, TIF file, 0.6 MB. Copyright © 2021 Chern et al. 2021 Chern et al. https://creativecommons.org/licenses/by/4.0/ This content is distributed under the terms of the Creative Commons Attribution 4.0 International license.

In agreement with its negative phenotype score, the knockout parasites grew poorly, which we assessed via plaque assay. These experiments showed a 69% reduction in plaque size, which was fully rescued by complementation (Fig. 2E). To address phosphorylation of ISAP1, we additionally complemented the Δ*isap1* strain with a phosphomutant copy in which 7 phosphosites identified by phosphoproteomics were mutated to alanine. This phosphomutant localized to similar spots and fully rescued the plaque defect, demonstrating that these phosphosites are not important for function (Fig. 2E; Fig. S3). In addition to plaque size, we observed an 80% reduction in plaque efficiency, which was mostly rescued by complementation (Fig. 2F). The reductions in plaque size and efficiency indicated that some element of the lytic cycle is disrupted in the Δ*isap1* strain. To further characterize the defect, we individually examined host cell invasion, intracellular replication, and egress. While no defect was observed in egress, we did see a significant loss of invasive capability of the knockout (Fig. 2G and H). We next examined replication and noticed that the Δ*isap1* parasites often appeared swollen and many vacuoles had misshapen parasites, more than two daughter buds, or a loss of coordinated endodyogeny (Fig. 2I to K). We also examined an array of other organelles, including the mitochondrion, apicoplast, rhoptries, micronemes, and endosome-like compartment (ELC), and found that they were mostly unaffected except in parasites with the most severe morphological problems, which suggested that these were the result of parasite death (Fig. S4).

10.1128/mBio.02455-21.4 FIG S4 Organelles unaffected in Δ*isap1* parasites. (A to E) IFA showing that many organelles are largely unaffected in Δ*isap1* parasites, including the mitochondrion (F1B-ATPase), apicoplast (ATrx1), rhoptries (ROP7), micronemes (MIC2), and ELC (NHE3). IMC6 is used to outline the periphery of the parasites. Magenta, organellar markers; green, rabbit anti-IMC6). Bars = 2 μm. Download FIG S4, TIF file, 1.6 MB. Copyright © 2021 Chern et al. 2021 Chern et al. https://creativecommons.org/licenses/by/4.0/ This content is distributed under the terms of the Creative Commons Attribution 4.0 International license.

### Deletion of ISAP1 affects cytoskeletal integrity and targeting of a subset of the cytoskeletal suture proteins.

**FIG 3:**
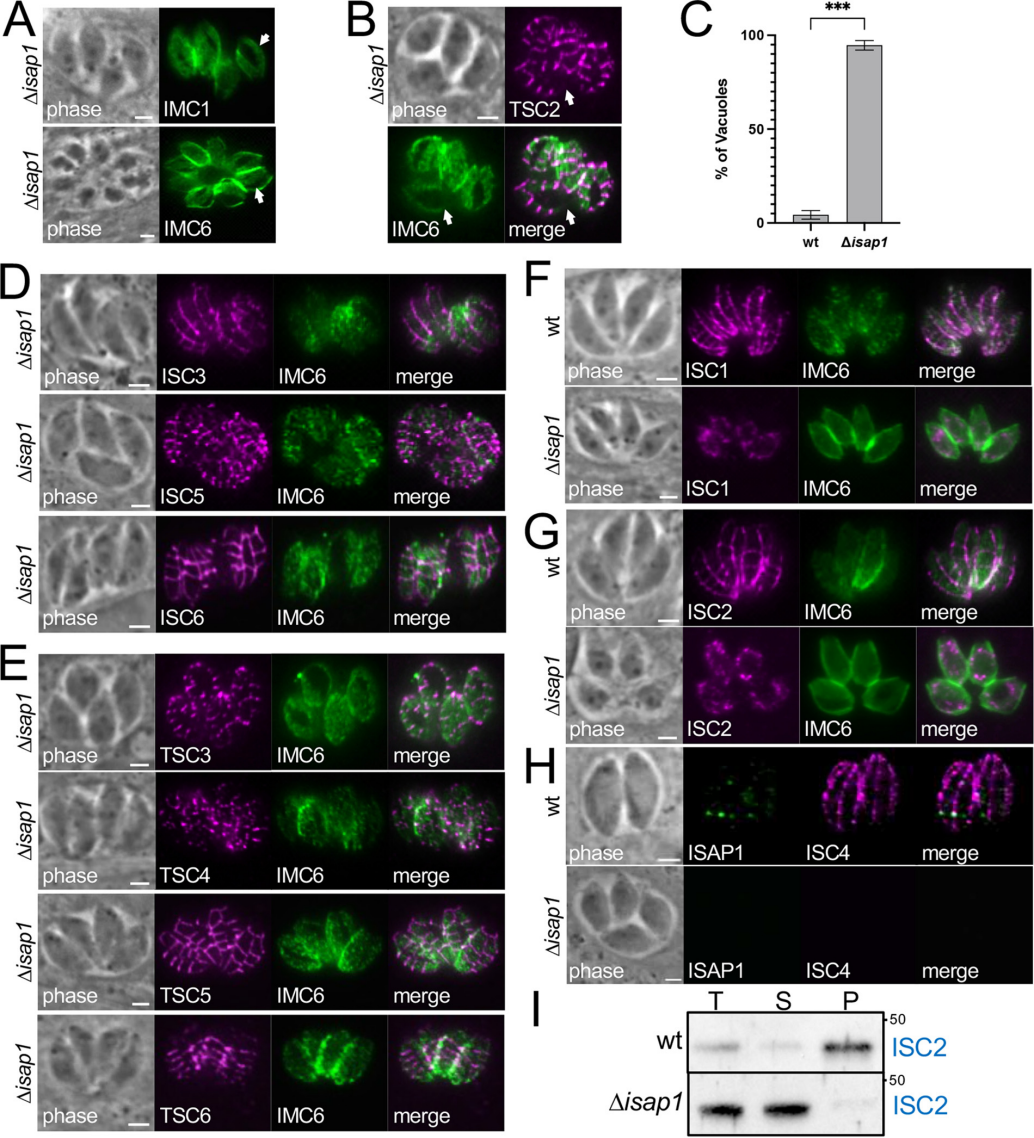
Loss of ISAP1 affects the integrity of the IMC cytoskeleton and results in mistargeting of a subgroup of cytoskeletal IMC suture proteins. (A) IFA of IMC1 and IMC6 in Δ*isap1* parasites showing gaps in the IMC cytoskeleton (arrows). The more internal IMC1/IMC6 staining pattern throughout the figure is due to the planes imaged for visualizing the gaps or sutures at the periphery of the parasite. Green, mouse anti-IMC1 and rabbit anti-IMC6. (B) IFA with the cytoskeletal transverse sutures protein TSC2 also shows the gaps in the Δ*isap1* cytoskeleton (arrows). TSC2 correctly targets the transverse sutures. Magenta, mouse anti-HA; green, rabbit anti-IMC6. (C) Quantification of the IMC6 cytoskeletal gaps showing 94.7% of Δ*isap1* vacuoles have parasites with gaps (***, *P < *0.001). (D) IFA of Δ*isap1* parasites showing that HA-tagged ISC3, ISC5, and ISC6 correctly target the sutures. Magenta, mouse anti-HA; green, rabbit anti-IMC6. (E) IFA of Δ*isap1* parasites showing that HA-tagged TSC3 and -4, Ty-tagged TSC5, and Myc-tagged TSC6 also correctly target the sutures. Magenta, mouse anti-HA, mouse anti-Ty, mouse anti-Myc; green, rabbit anti-IMC6. (F and G) IFA showing that ISC1 and ISC2 are mislocalized to cytoplasmic spots in Δ*isap1* parasites. Magenta, rat anti-ISC1, rat anti-ISC2; green, rabbit anti-IMC6. (H) Disruption of *ISAP1* in an ISAP1-smOLLAS- and ISC4-HA-tagged background results in the loss of ISC4. Magenta, mouse anti-HA (ISC4); green, rabbit anti-IMC6. (I) Western blot analysis of TX-100 detergent fractionation shows that ISC2 is no longer associated with the cytoskeleton in Δ*isap1* parasites. T, total; S, detergent-soluble supernatant; P, detergent-insoluble cytoskeletal pellet. Bars = 2 μm.

In the course of examining the morphological defects of the Δ*isap1* parasites, we also noticed that many of the parasites had an apparent gap in the alveolin network proteins IMC1 and IMC6, suggesting a breach in the cytoskeleton (Fig. 3A). To further explore this and to begin to examine the IMC suture proteins, we tagged the cytoskeletal suture protein TSC2 in Δ*isap1* parasites, which correctly targeted to the sutures but also showed an apparent breach of the cytoskeleton (Fig. 3B). This gap was present in nearly all (94.7%) of the Δ*isap1* vacuoles examined (Fig. 3C). We then examined the localization of several other suture proteins, including ISC1 to -6 and TSC3 to -6. While ISC3, -5, and -6 and TSC3 to -6 appeared to traffic to the sutures correctly (Fig. 3D and E), ISC1 and ISC2 were mislocalized to a cytoplasmic spotted pattern (Fig. 3F and G), and ISC4 was absent altogether, even though tagging was verified by PCR (Fig. S5). To confirm that the absence of ISC4 was not due to some artifact of the tagging in Δ*isap1* parasites, we disrupted *ISAP1* in an ISAP1-smOLLAS- and ISC4-3×HA-tagged background and observed the loss of ISC4-3×HA upon disruption of *ISAP1* (Fig. 3H). To examine if the mislocalized proteins were tethered to the cytoskeleton, we conducted detergent fractionation assays on Δ*isap1* parasites. While our ISC1 antibody was insufficient to detect the protein following dilution for fractionation, ISC2 was clearly no longer tethered to the cytoskeleton and instead efficiently solubilized upon detergent fractionation (Fig. 3I). Together, these data indicate that ISAP1 is necessary for the correct targeting of ISC1, -2, and -4 and for the integrity of the cytoskeletal meshwork of the IMC.

10.1128/mBio.02455-21.5 FIG S5 Disruption of *ISAP1* results in loss of ISC4. (A) IFA showing that ISC4 is absent in Δ*isap1* parasites. ISC4 was endogenously 3×HA tagged in both wild-type (wt) and Δ*isap1* parasites. Magenta, mouse anti-HA (ISC4); green, rabbit anti-IMC6. Bars = 2 μm. (B) Strategy and agarose gel analysis showing PCR verification of correct tagging of ISC4 in Δ*isap1* parasites. Download FIG S5, TIF file, 0.6 MB. Copyright © 2021 Chern et al. 2021 Chern et al. https://creativecommons.org/licenses/by/4.0/ This content is distributed under the terms of the Creative Commons Attribution 4.0 International license.

### The N-terminal region of ISAP1 is important for localization and function.

**FIG 4:**
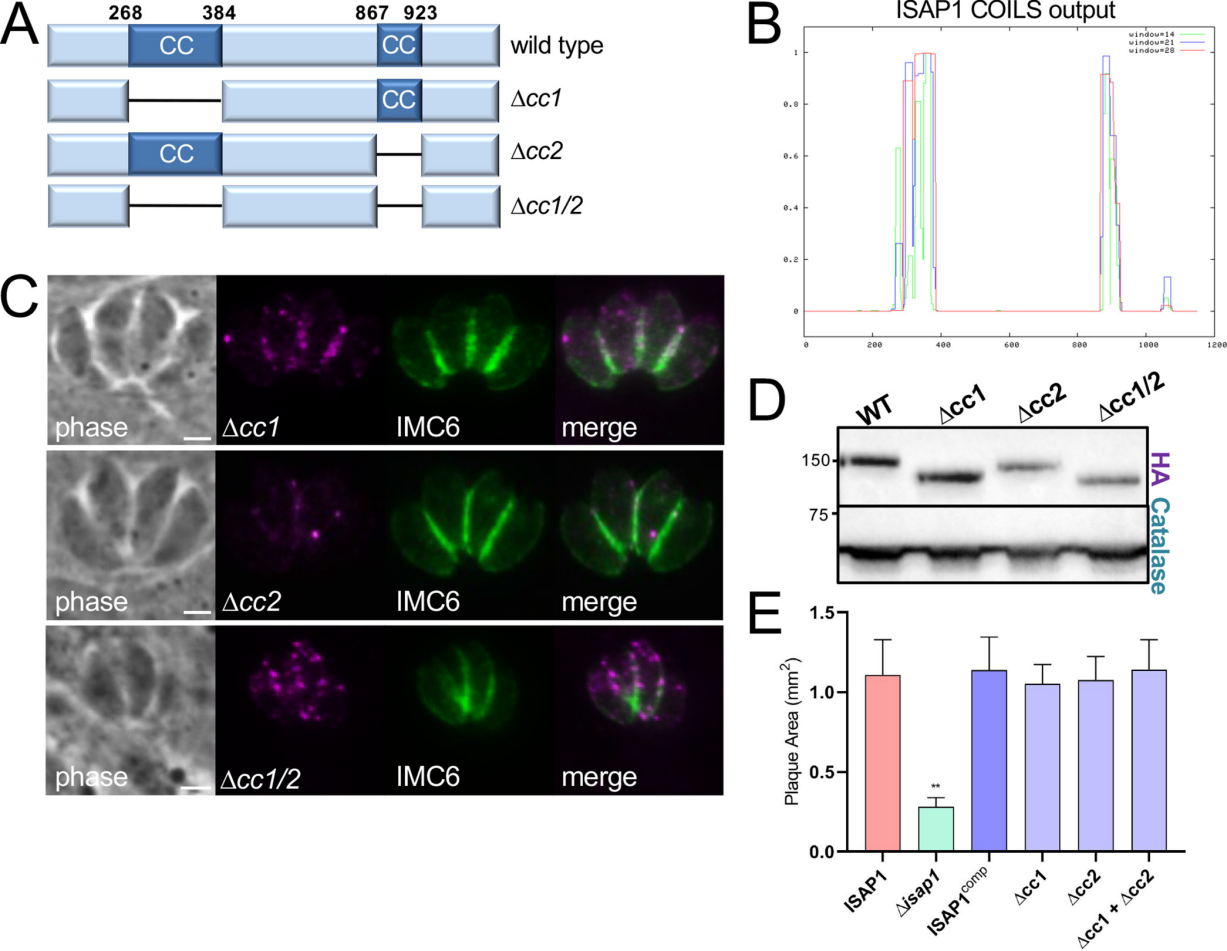
The CC domains of ISAP1 are not required for function. (A) Diagram showing deletions of the CC domains individually or together. (B) ISAP1 CC domains predicted by the COILS server (43). (C) IFA showing that the 3×HA-tagged CC deletions localize to puncta similarly to wild-type ISAP1. Magenta, mouse anti-HA; green, rabbit anti-IMC6. Bars = 2 μm. (D) Western blot analysis comparing expression levels of 3×HA-tagged ISAP1^comp^ to the CC deletions. (E) Plaque assays showing that ISAP1 CC deletion constructs can fully rescue the plaque defect of Δ*isap1* parasites (**, *P < *0.01).

Aside from its two CC domains (Fig. 4A and B), ISAP1 lacks homology to known proteins or domains that would provide a clue to its function. We have previously shown the importance of CC domains in the early daughter protein IMC32 and the IMC cytoskeletal protein ILP1 (21, 24). To determine if the ISAP1 CC domains are important for targeting or function, we deleted each of the domains individually or together and expressed the deletion constructs in Δ*isap1* parasites (Fig. 4A). All of the deletions localized to a series of faint spots similar to that seen for the endogenously tagged protein (Fig. 4C). Some slight variability in the expression levels of the deletion constructs was observed (Fig. 4D); however, all of them were fully able to rescue the knockout, as assessed by plaque assays (Fig. 4E). These results demonstrate that the CC domains of ISAP1 are dispensable for its localization and function.

**FIG 5:**
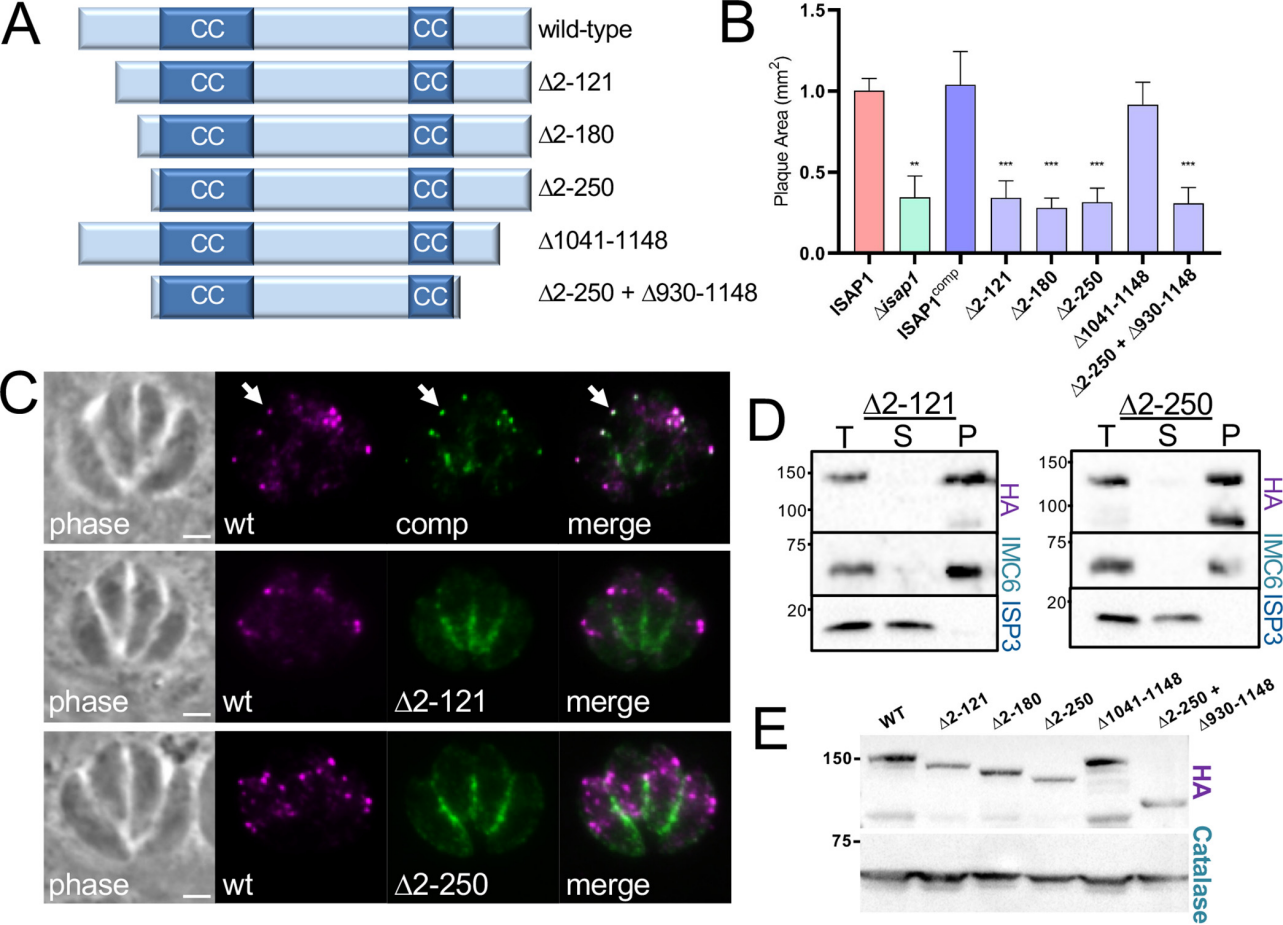
The N-terminal region of ISAP1 is important for punctum trafficking and function. (A) Diagram showing the N- and C-terminal deletion constructs. (B) Plaque assays showing that the conserved C-terminal region of ISAP1 can be deleted but the N-terminal 121 amino acids of the protein is important for function (**, *P < *0.01; ***, *P < *0.001). (C) IFA showing that the full-length smMyc-tagged ISAP1 complement (comp) targets puncta similarly to the endogenously smHA-tagged protein (wt) (arrows). Loss of the N-terminal 121 or 250 amino acids of ISAP1 results in relocalization to the periphery of the parasite, consistent with IMC localization. Magenta, rabbit anti-HA; green, mouse anti-Myc. Bars = 2 μm. (D) Detergent fractionation showing that 3×HA-tagged Δ2–121 and Δ2–250 constructs still associate with the cytoskeleton. ISP3 and IMC6 were used as controls for the membrane and cytoskeletal fractions, respectively. T, total; S, detergent-soluble supernatant; P, detergent-insoluble cytoskeletal pellet. (E) Western blot analysis comparing expression levels of 3×HA-tagged ISAP1^comp^ to those of proteins with N- and C-terminal deletions.

We then focused on the N-terminal region upstream of the first CC domain (residues 1 to 250) and the C-terminal region downstream of the second CC domain (residues 1041 to 1148), as these portions have higher regions of homology, suggesting that they may contain functional domains (Fig. 5A; Fig. S1). While the C-terminal deletion construct rescued the knockout, absence of the N-terminal region (Δ2–250) eliminated the ability to rescue function (Fig. 5B). To further dissect the functional region of the N-terminal domain, we made two additional smaller deletions that removed residues 2 to 121 or 2 to 180, each of which resulted in the loss of regions with significant homology (Fig. S1). Neither of these constructs was able to rescue the phenotype of the knockout, demonstrating that the N-terminal 121 amino acids are essential for ISAP1 function (Fig. 5B). To determine if this was due to a loss of trafficking, we expressed the Δ2–121 and Δ2–250 ISAP1 constructs with a smMyc tag in wild-type parasites in which endogenous ISAP1 was tagged with smHA. IFA analysis showed that the deletion constructs failed to localize to the puncta but instead localized primarily to the periphery of the parasite, consistent with IMC localization (Fig. 5C). Detergent extraction experiments using 3×HA-tagged versions of the deletions showed that these proteins still associate with the cytoskeleton, demonstrating that these regions are not necessary for cytoskeletal tethering (Fig. 5D). We again observed some variability in expression levels of the deletions (Fig. 5E), but all were similar to those seen with the CC deletions, which fully rescue the ISAP1 knockout (Fig. 4D). ISAP1 also contains a weakly predicted palmitoylation site at residue 87 (C87), which is in the critical N-terminal region of the protein. To determine if palmitoylation at this site is responsible for ISAP1 targeting or function, we mutated C87 to serine and found that the ISAP1^C87S^ mutant was able to localize to puncta and rescue the ISAP1 knockout, similar to the wild-type protein (Fig. S6). Together, these data demonstrate that the N-terminal 121 amino acids of ISAP1 are required for suture punctum localization and function but not for association with the IMC cytoskeleton.

10.1128/mBio.02455-21.6 FIG S6 Mutagenesis of the ISAP1 predicted palmitoylation site (C87S) does not affect ISAP1 targeting or function. (A) IFA showing that smHA-tagged ISAP1^C87S^ localizes to puncta that colocalize with smOLLAS-tagged wild-type ISAP1. Magenta, rat anti-OLLAS; green, rabbit anti-HA. Bar = 2 μm. (B) Plaque assay showing that ISAP1^C87S^ rescues the Δ*isap1* strain similarly to the wild-type complemented strain (ISAP1^comp^) (**, *P < *0.01). Download FIG S6, TIF file, 0.4 MB. Copyright © 2021 Chern et al. 2021 Chern et al. https://creativecommons.org/licenses/by/4.0/ This content is distributed under the terms of the Creative Commons Attribution 4.0 International license.

### *In vivo* biotinylation reveals candidate ISAP1-interacting proteins.

**FIG 6:**
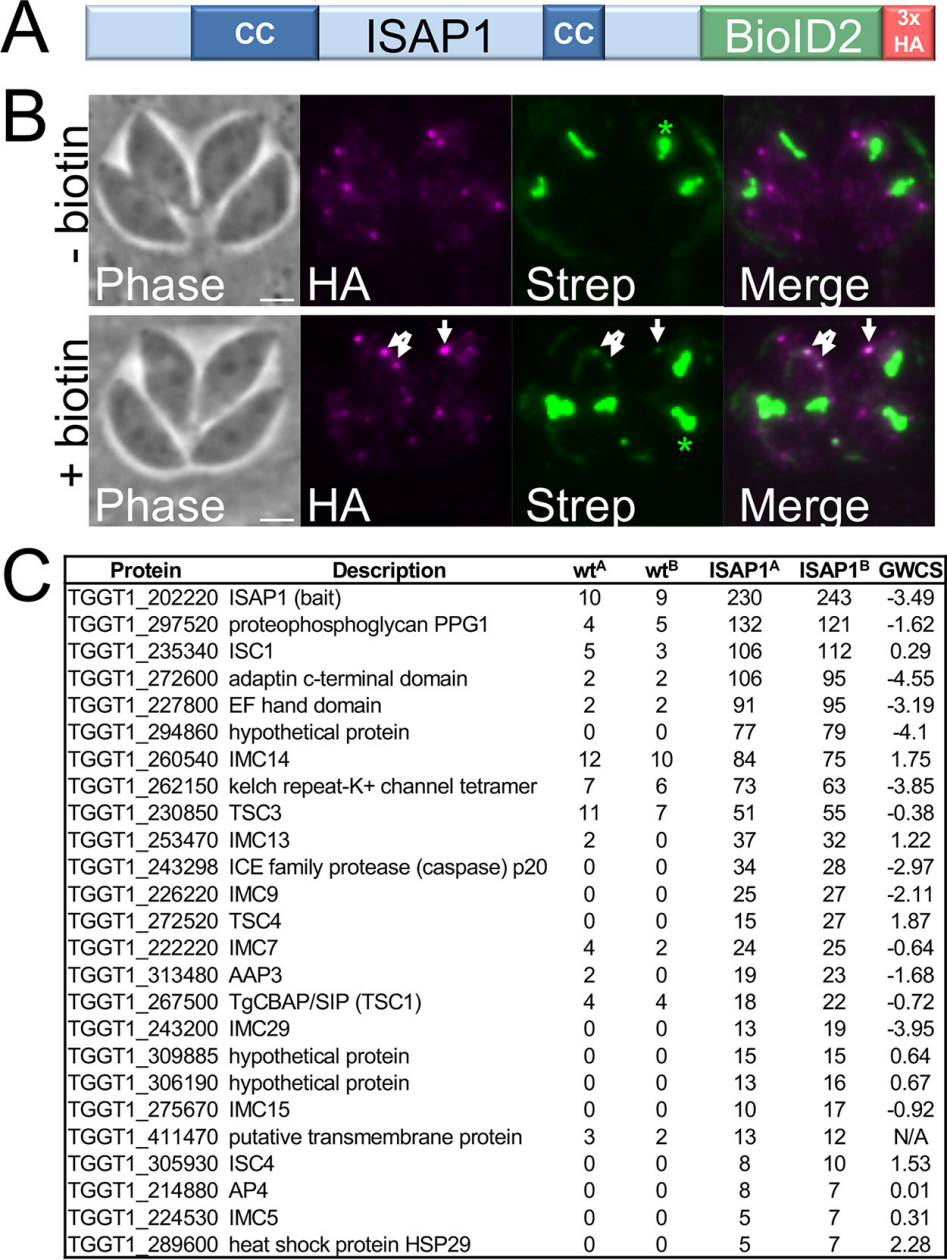
*In vivo* biotinylation using ISAP1 as bait identifies candidate interacting proteins. (A) Diagram showing the ISAP1-BioID2 fusion generated by endogenous gene tagging. A 3×HA tag is included for detection of the fusion protein. (B) IFA showing that the ISAP1-BioID2 fusion targets puncta similarly to the wild-type protein and is active, as assessed by faint streptavidin staining upon addition of biotin to the medium (arrows). Asterisks indicate the endogenously biotinylated signal in the apicoplast. Magenta, mouse anti-HA; green, streptavidin 488. Bars = 2 μm. (C) Table showing the top 25 hits from streptavidin purification of ISAP1-BioID2 following detergent fractionation of the cytoskeleton. Two replicates were performed (replicates are labeled A and B), and untagged parasites plus biotin were used as the control. Spectral counts are shown for each sample. GWCS, phenotype score assigned in a genome-wide CRISPR/Cas9 screen (22).

To better understand how ISAP1 is tethered to the parasite’s cytoskeleton and functions in invasion and replication, we carried out *in vivo* biotinylation (using BioID2) with ISAP1 as a bait protein (Fig. 6A) (25). The endogenously tagged ISAP1-BioID2 fusion protein localized to spots in the cytoplasm similarly to the HA-tagged protein (Fig. 6B). The fusion was also active as assessed by streptavidin staining upon the addition of biotin to the medium, although the staining level was low, in agreement with the weak detection of the endogenously tagged bait protein. To identify candidate interacting proteins, we performed a large-scale ISAP1-BioID2 *in vivo* biotinylation experiment. We showed previously with the cytoskeletal IMC suture protein ISC4 that detergent fractionation can dramatically reduce background (13); therefore, we included this step in the ISAP1-BioID2 experiment and analyzed the purified proteins via mass spectrometry.

Confirming the activity of the bait protein, ISAP1 was the top hit identified by mass spectrometry (Fig. 6C). In addition, 14 of the top 25 hits were known IMC proteins, including IMC4, -5, -7, -9, -13, -14, -15, and -29, the IMC suture proteins ISC1, ISC4, TSC1 (CBAP, SIP), TSC3, and TSC4, and the apical annuli protein AAP3. Also highly ranked in this data set were TGGT1_297520, which is annotated as proteophosphoglycan 1 (PPG1), and two proteins which have been implicated in vesicle transport, the EPS15/intersectin-1-like protein TGGT1_227800 and the putative AP-2 adaptor complex member TGGT1_272600 (26). TGGT1_297520 (PPG1) was previously reported to be undetectable in tachyzoites but localizes to cytoplasmic spots plus the parasitophorous vacuole in bradyzoites (27). The localization of TGGT1_227800 has not been shown, but TGGT1_272600 reportedly localizes to low-abundance cytoplasmic puncta that colocalize with the dynamin-related vesicle trafficking protein DrpC (although this data was not shown) (26, 27). These results together suggest potential links between ISAP1 and the IMC cytoskeleton and identify several candidate interactors in the IMC puncta.

### Verification of BioID hits identifies proteins that colocalize with ISAP1.

**FIG 7:**
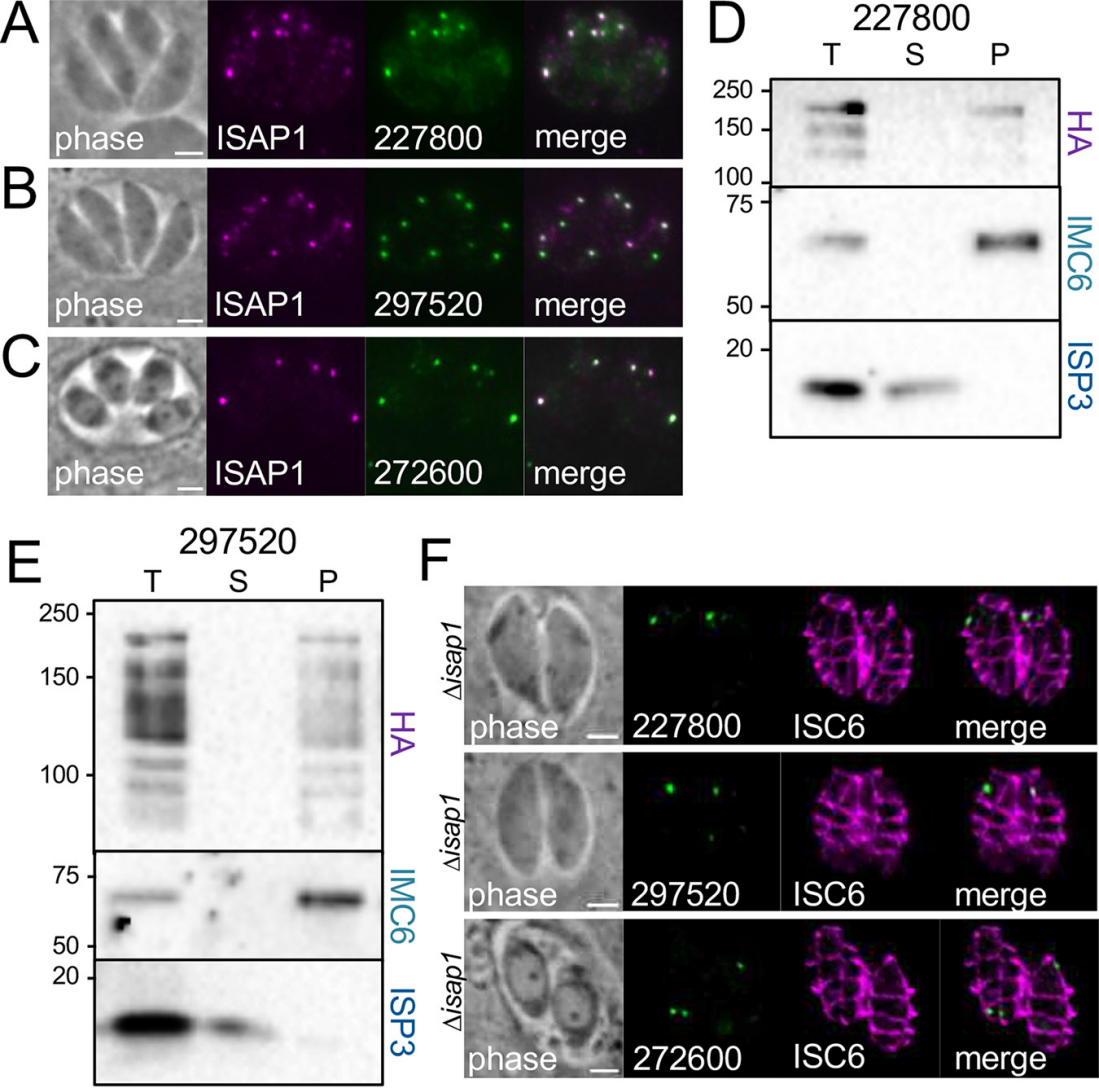
Endogenous tagging of candidates reveals proteins that colocalize with ISAP1. (A) IFA showing the EPS15/intersectin-1-like protein TGGT1_227800-smOLLAS localizes to discrete spots that colocalize with ISAP1-smHA. Magenta, rabbit anti-HA; green, rat anti-OLLAS. (B) IFA showing that TGGT1_297520-3×HA also colocalizes with the ISAP1-smOLLAS puncta. Magenta, rat anti-OLLAS; green, mouse anti-HA. (C) The AP2 adaptor complex member TGGT1_272600-3×HA also colocalizes with ISAP1-smOLLAS. Magenta, rat anti-OLLAS; green, mouse anti-HA. (D and E) Detergent fractionation shows that TGGT1_227800 and TGGT1_297520 are tethered to the cytoskeleton, although both of the proteins are labile and suffered substantial reproducible breakdown during fractionation. ISP3 and IMC6 were used as controls as described above. (F) IFA showing that 3×HA-tagged TGGT1_227800, TGGT1_297520, and TGGT1_272600 all retain their suture punctum localization in Δ*isap1* parasites. Green, mouse anti-HA; magenta, rabbit anti-Myc detecting 3×Myc-tagged ISC6. Bars = 2 μm.

To determine if TGGT1_297520 (PPG1), TGGT1_227800 (EPS15/intersectin-1-like), and TGGT1_272600 (AP-2) represent likely ISAP1 partners, we used CRISPR/Cas9 to endogenously tag their genes in a spaghetti monster ISAP1-tagged background (23, 28). IFA analysis showed that TGGT1_227800, TGGT1_297520, and TGGT1_272600 all localized in cytoplasmic spots that colocalized well with ISAP1 (Fig. 7A to C). To determine if these proteins are associated with the cytoskeleton, we conducted detergent extraction experiments as described above for ISAP1. While we were unable to detect TGGT1_272600 in the diluted conditions required for fractionation, both TGGT1_297520 and TGGT1_227800 fractionated primarily with the cytoskeletal fraction, although both proteins reproducibly suffered significant breakdown during fractionation (Fig. 7D and E). The colocalization and cytoskeletal cofractionation of these players agree with their high ranking in the BioID experiment and suggest that they may interact with ISAP1 at this location. While this group of proteins colocalize with ISAP1 in the sutures, they are not dependent on ISAP1 for localization at this site, as they each retain their suture punctum localization in Δ*isap1* parasites (Fig. 7F).

### The vesicle trafficking protein DrpC partially colocalizes with ISAP1 and largely fractionates with the cytoskeleton.

**FIG 8:**
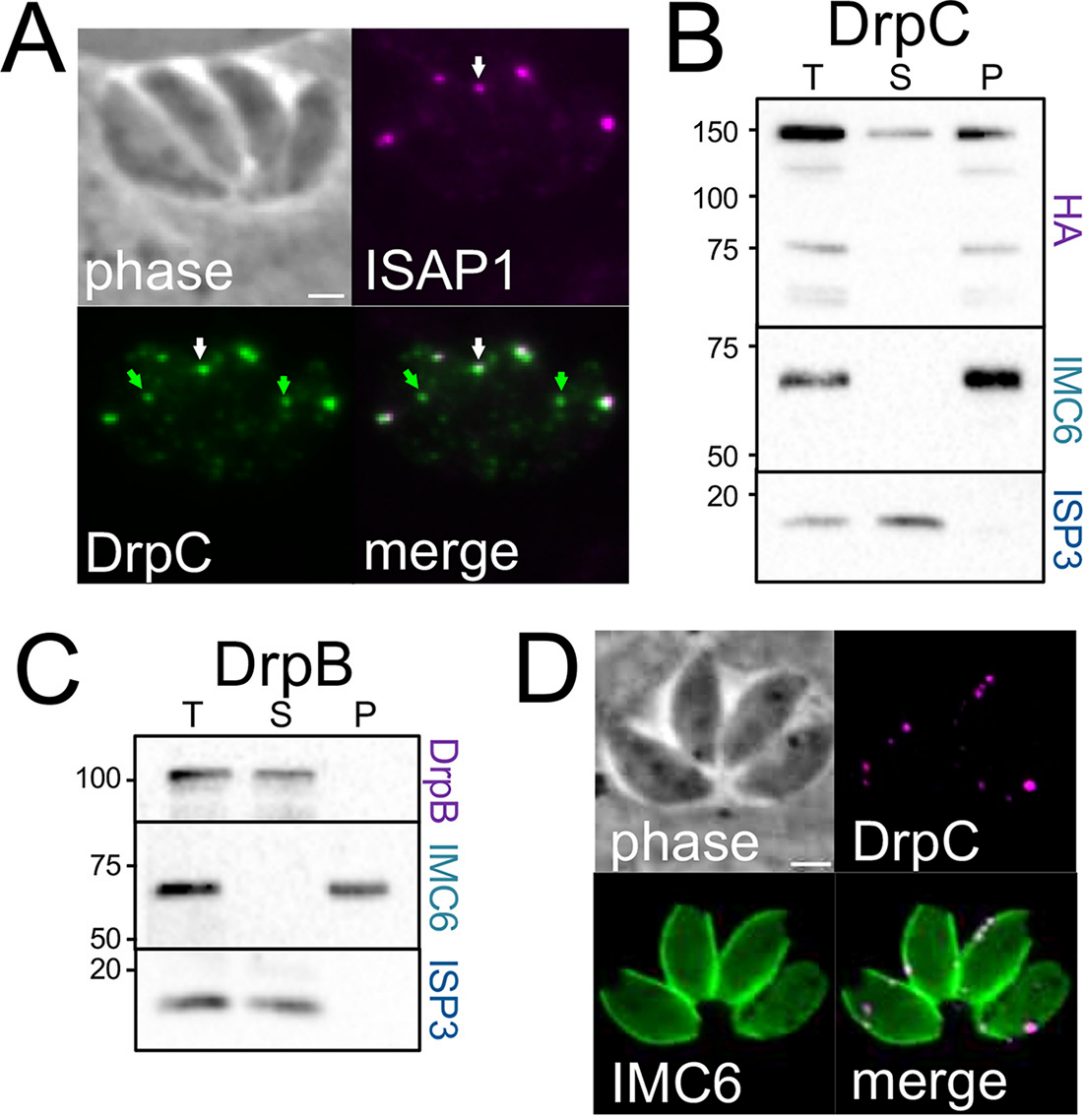
The membrane trafficking protein DrpC colocalizes with ISAP1 and associates with the cytoskeleton. (A) IFA showing that 3×HA-tagged DrpC mostly colocalizes with ISAP1-smOLLAS (white arrows), though unique DrpC spots are also present (green arrows). Magenta, rat anti-OLLAS; green, rabbit anti-HA. (B) Detergent fractionation shows that a substantial portion of DrpC is surprisingly tethered to the cytoskeleton. ISP3 and IMC6 were used as controls as described above. (C) The related dynamin-related protein DrpB is readily solubilized by detergent extraction. (D) IFA showing that 3×HA-tagged DrpC retains its cytoplasmic punctum localization in Δ*isap1* parasites. Magenta, mouse anti-HA; green, rabbit anti-IMC6. Bars = 2 μm.

TGGT1_227800 and TGGT1_272600 were previously identified as coprecipitating proteins with the vesicle trafficking protein DrpC, which also localizes to a series of cytoplasmic spots in the parasite (26). While DrpC was not identified in our BioID experiments (and ISAP1 was not identified in the DrpC pulldown), its similar localization pattern and common partners suggested that it may also colocalize with ISAP1. We thus assessed DrpC colocalization in the ISAP1-smOLLAS-tagged strain. Most of the DrpC puncta colocalize with ISAP1, but additional spots were also observed (Fig. 8A). Detergent fractionation also demonstrated that a substantial portion of DrpC partitions with the cytoskeleton, although some is released by detergent extraction (Fig. 8B), further linking trafficking elements to ISAP1 puncta in the parasite’s cytoskeleton. We were surprised that DrpC and other putative trafficking proteins were tethered to the cytoskeleton; therefore, we also examined the related protein DrpB (29), and found that it was efficiently released into the detergent-soluble fraction, as expected (Fig. 8C). Similar to the other putative interactors, the cytoplasmic punctum localization of DrpC appears unaffected in Δ*isap1* parasites, indicating that other cellular components participate in the organization of vesicle trafficking machinery at the IMC suture puncta (Fig. 8D). Together, these data demonstrate that ISAP1 is an important component of the suture puncta and suggests that the puncta are a site of trafficking of cargo across the IMC.

## DISCUSSION

In this paper, we identify and characterize ISAP1, a new component of the T. gondii IMC that localizes to distinct cytoskeletal puncta that are embedded in the sutures of the organelle. Low expression levels made our initial characterization of the protein difficult to interpret, but this was resolved using high-sensitivity spaghetti monster tags that enabled better detection of the protein (20, 28). The increase in ISAP1 expression during daughter bud formation suggests that the puncta are synthesized and assembled onto the forming daughter buds with a “just-in-time” approach, as is seen for other IMC proteins (11). This spot-like pattern anchored in the sutures thus represents a new type of localization within the IMC.

Like most IMC proteins, ISAP1 lacks homology to known proteins or identifiable domains other than its CC domains. CCs are structural motifs that consist of alpha helices that are coiled together and often function in oligomerization or protein-protein interactions (18). We and others showed previously that CCs play important roles in the IMC for the cytoskeletal protein ILP1 and the early daughter membrane protein IMC32, and these motifs are also frequently present in the constituents of the conoid and apical annuli in T. gondii (15, 17, 21, 24). Surprisingly, deletion of the ISAP1 CCs individually or together demonstrated that they are dispensable for localization and function. However, our deletion analyses revealed a conserved domain in the N-terminal 121 amino acids that plays a critical role in both localization and function. While loss of this region disrupts punctum localization, the deletion protein retains tethering to the IMC cytoskeleton. Together, our deletion analyses suggest that IMC cytoskeletal binding is likely conferred by the central region of the protein between the CC domains (or by residues 923 to 1041 downstream of the second CC), with the N-terminal region being responsible for tethering to the puncta, perhaps via binding to another protein at this location.

Our BioID experiments revealed a number of candidate interactors that may mediate binding to the IMC cytoskeleton or puncta organization. The IMC suture proteins ISC1, ISC4, TSC1, TSC3, and TSC4 were highly ranked in the BioID experiment and may represent cytoskeletal attachment points on the longitudinal or transverse sutures (12). The array of known IMC proteins identified (e.g., IMC7, -9, -13, and -14) may represent additional contact points that tether ISAP1 to the cytoskeleton. The small plaque size and replication defects of Δ*isap1* parasites are reminiscent of the knockout of the suture protein ISC3, although ISC3 is membrane associated and not cytoskeletal like ISAP1 (13). While ISC3 localization does not appear to be impacted in Δ*isap1* parasites, the loss of the cytoskeletal proteins ISC1, -2, and -4 may impact the ability of ISC3 or other membrane suture proteins to be properly tethered to the cytoskeleton (12, 13, 23). Loss of ISAP1 also appears to impact the structure or integrity of the cytoskeleton, which likely results in the morphological changes leading to replication and invasion defects. The precise means by which ISAP1 controls parasite shape is likely to be best understood by determining how the protein acts with its interactors within the suture puncta.

One of the top BioID hits that colocalizes with ISAP1 and fractionates with the cytoskeleton was TGGT1_297520, which is annotated the putative phosphoproteoglycan PPG1 (27). However, its similarity to phosphoproteoglycans is not clearly supported by BLAST or the OrthoMCL database (19, 30). Intriguingly, the protein contains a C-terminal GAR domain, which is involved in microtubule binding and could confer binding to the subpellicular microtubules or perhaps to the alveolins that form the IMC cytoskeleton (31). In addition, the protein structure-based prediction program iTasser suggests similarity to the cytoskeletal protein talin, which is known to interact with vimentin, an intermediate filament protein like the alveolins (32, 33). Also identified were the membrane trafficking-implicated proteins TGGT1_227800 (EPS15/intersectin-1-like) and TGGT1_272600 (AP-2 complex member), which were previously identified in DrpC pulldowns (26). The association with membrane trafficking proteins suggests that they may function in the delivery of cargo into or across the IMC. The association with the AP-2 adaptor complex, EPS15/intersectin-1-like proteins, and DrpC could also suggest a role in endocytosis at this site. Endocytosis is still poorly understood in T. gondii, but it is believed to occur at a structure called the micropore (26, 34). The IMC suture puncta seem likely to correlate with the micropore, but additional experiments will be necessary to confirm this and a direct role in endocytosis at this site.

While DrpC colocalizes partially with ISAP1 in the IMC suture puncta, it was not identified in our ISAP1-BioID2 experiment. This may indicate that it is more distantly located in the puncta or that biotinylation was impeded by the other colocalizing proteins, which may serve as a bridge between ISAP1 and DrpC. Because we used detergent fractionation to enrich for cytoskeletal factors, it is also possible that other interacting or proximal proteins were missed due to the fractionation, which would best be evaluated using whole-parasite extracts and TurboID for improved proximity labeling (35).

Overall, our data agree perfectly with a recent study that demonstrated that DrpC localizes in a spot-like pattern similar to ISAP1, associates with membrane trafficking proteins, and regulates IMC structure (26). DrpC has additionally been shown to function in mitochondrial fission at the end of replication (36). It is possible that DrpC is able to perform multiple functions such as mitochondrial fission via its localization to additional puncta within the parasite. Together, these studies indicate that these proteins serve as a portal for trafficking of material across the IMC at discrete points along the IMC sutures. While the vesicle trafficking machinery colocalizes with ISAP1, it is not necessary for their localization to the puncta. However, this does not exclude the possibility that loss of ISAP1 impacts the function of the trafficking machinery at this site. Further dissection of ISAP1, DrpC, and each of their interacting partners will provide a deeper understanding into how these proteins regulate cellular functions in T. gondii.

## MATERIALS AND METHODS

### *Toxoplasma* and host cell culture.

T. gondii RHΔ*ku80*Δ*hpt* and modified strains were grown on confluent monolayers of human foreskin fibroblast (HFF) host cells in Dulbecco’s modified Eagle medium (DMEM) supplemented with 10% fetal bovine serum, as previously described (37).

### Antibodies.

The following previously described primary antibodies were used in immunofluorescence (IFA) or Western blot assays: mouse anti-ISP1 (38), mouse anti-ISP3 (39), rabbit anti-IMC6 (24), mouse anti-F1β subunit (monoclonal antibody [MAb] 5F4) (40), mouse anti-ATrx1 (MAb 11G8) (41), mouse anti-ROP7 (42), mouse anti-MIC2 (43), rabbit anti-catalase (44), and guinea pig anti-NHE3 (45). The hemagglutinin (HA) epitope was detected with mouse anti-HA (MAb HA.11) (BioLegend) or rabbit anti-HA (Invitrogen). The c-Myc epitope was detected with mouse anti-Myc (MAb 9E10), and the OLLAS tag was detected using rat monoclonal anti-OLLAS (28). For production of ISC1 and ISC2 antibodies, the complete coding sequences of the genes were cloned into the pET28 and pET160 bacterial expression vectors, respectively. The constructs were transformed into BL21(DE3) E. coli, and proteins were induced with 1 mM IPTG (isopropyl-β-d-thiogalactopyranoside) and purified using nickel-nitrilotriacetic acid (Ni-NTA) agarose under denaturing conditions as described elsewhere (46). The samples were then dialyzed into phosphate-buffered saline (PBS) to remove the urea, and rat antisera against the proteins were produced by Cocalico Biologicals.

### IFA and Western blotting.

For IFA, HFFs were grown to confluence on coverslips and infected with T. gondii parasites. After 18 to 36 h, the coverslips were fixed and processed for indirect immunofluorescence using either 3.7% formaldehyde or 100% ice-cold methanol as previously described (46). Primary antibodies were detected by species-specific secondary antibodies conjugated to Alexa Fluor 594/488. The coverslips were mounted in Vectashield (Vector Labs) and viewed with an Axio Imager.Z1 fluorescence microscope (Zeiss) as described elsewhere (42).

For Western blotting, parasites were lysed in Laemmli sample buffer (50 mM Tris-HCl [pH 6.8], 10% glycerol, 2% SDS, 0.1 M dithiothreitol [DTT], 0.1% bromophenol blue) and lysates were resolved by SDS-PAGE and transferred onto nitrocellulose membranes. Blots were probed with the indicated primary antibodies, followed by secondary antibodies conjugated to horseradish peroxidase (HRP). Target proteins were visualized by chemiluminescence (Thermo Scientific).

### Epitope tagging.

For endogenous tagging of proteins, we used CRISPR/Cas9 as previously described (16, 23). The appropriate guides were ligated into the pU6 Universal plasmid and the epitope tag plus selectable marker was amplified from LIC (ligation-independent cloning) epitope-tagging plasmids (e.g., 3×HA, 3xMyc, smHA, smOLLAS, and smMyc) with 40-bp flanking regions for recombination at the 3′ end of each gene. Following transfection of the guide plus PCR product, transgenic parasites were selected in the appropriate drug medium (containing either 1 μM pyrimethamine, 50 μg/ml mycophenolic acid/xanthine, or 1 μM chloramphenicol) and cloned by limiting dilution. Clones that had undergone the intended recombination event were screened by IFA and Western blotting against the epitope tag. Tagging of TGGT1_248740, ISC5, and TSC2 to -6 was performed using the LIC method as previously described (13). The PCR verification of ISC4 tagging in the Δ*isap1* parasites was performed with primers p64 to p66 (Table S1).

10.1128/mBio.02455-21.7 TABLE S1 Oligonucleotide primers used in this study. All primer sequences are shown in the 5′-to-3′ orientation. Download Table S1, PDF file, 0.07 MB. Copyright © 2021 Chern et al. 2021 Chern et al. https://creativecommons.org/licenses/by/4.0/ This content is distributed under the terms of the Creative Commons Attribution 4.0 International license.

### Detergent extractions.

Extracellular parasites were washed in PBS, pelleted, and lysed in 1 ml of 1% Triton X-100 lysis buffer (50 mM Tris-HCl [pH 7.4], 150 mM NaCl) supplemented with Complete protease inhibitor cocktail (Roche) for 20 min on ice. Lysates were centrifuged for 10 min at 16,000 × *g* at 4°C. Equivalent amounts of total, supernatant (detergent-soluble), and pellet (detergent-insoluble) fractions were separated by SDS-PAGE and analyzed by Western blotting. IMC6 and ISP3 served as controls for the cytoskeletal and membrane fractions, respectively (24, 39).

### Gene knockout and phenotypic analyses.

CRISPR/Cas9 and homologous recombination were used to knockout the *ISAP1* gene, as previously described using primers p5 to p10 (47). Knockout clones were verified by IFA and PCR using primers p11 to p14.

For plaque assays, intracellular parasites were collected by scraping and passaging through a 27-gauge needle, and then equivalent parasite numbers were allowed to infect HFF monolayers. Seven days after infection, cells were fixed with 100% ice-cold methanol and stained with crystal violet (47). The area of 50 plaques per condition was measured using ZEN software (Zeiss). All plaque assays were performed in triplicate using biological replicates. Plaque number was counted manually to measure efficiency of plaquing. Statistical significance was calculated using unpaired *t* tests comparing each condition against the wild-type HA-tagged ISAP1.

For egress assays, parasites were grown on coverslips with HFF monolayers for 30 h, washed with warm HBSS, incubated with the calcium ionophore A23187 or DMSO control at 37°C for 3 min, and then fixed and stained, as previously described (9). The percentage of lysed vacuoles was counted for three replicates per condition.

For invasion assays, parasites were settled onto HFF monolayers on coverslips in warm Endo buffer for 20 min and then allowed to invade using warm D1 medium for 15 min, as previously described (9). Coverslips were fixed and blocked, and extracellular parasites were differentially stained before the cells were permeabilized and all parasites were stained, to record parasites as invading or not. Invasion assays were performed in triplicate, and statistical significance was calculated using unpaired *t* tests comparing each condition against the wild-type HA-tagged ISAP1.

### Complementation with wild-type and mutant constructs.

To generate the wild-type complement construct, the entire coding region of the gene was PCR amplified from genomic DNA and cloned into a uracil phosphoribosyltransferase (UPRT) locus knockout vector driven by the ISC6 promoter, as previously described (primers p15 to p18) (9). The phosphomutant was constructed using synthetic genes (Quintara Biosciences) to mutate each phosphosite to an alanine in the complementation vector. Deletion constructs were built with the complementation vector as a template using the Q5 site-directed mutagenesis kit to delete specific sequences (primers p19-p31). The plasmids were linearized and transfected into the Δ*isap1* parasites and selected with 5-fluoro-5′-deoxyuridine (FUDR). Parasites expressing the complementation constructs were cloned by limiting dilution.

### Affinity purification of biotinylated proteins.

HFF monolayers infected with parasites expressing the ISAP1-BioID2 fusion or the respective parental line were grown in media containing 150 μM biotin for 24 h prior to parasite egress. Approximately 10^9^ extracellular parasites were collected, washed in PBS, and lysed in radioimmunoprecipitation assay (RIPA) buffer (50 mM Tris [pH 7.5], 150 mM NaCl, 0.1% SDS, 0.5% sodium deoxycholate, 1% NP-40) supplemented with Complete protease inhibitor cocktail (Roche) for 30 min on ice. Lysates were centrifuged for 15 min at 16,000 × *g* to pellet insoluble debris and then incubated with high-capacity streptavidin agarose (Pierce) at room temperature for 4 h under gentle agitation. Beads were collected by centrifugation and washed five times in RIPA buffer, followed by three washes in 8 M urea buffer (8 M urea, 50 mM Tris-HCl [pH 7.4], 150 mM NaCl). Of each sample, 10% was boiled in Laemmli sample buffer, and eluted proteins were analyzed by Western blotting, while the remaining material was digested directly from the beads for mass spectrometry as described elsewhere (12).

### Biotinylated protein sample digestion and desalting.

The proteins bound to streptavidin beads were reduced and alkylated via sequential 20-min incubations of 5 mM TCEP [Tris(2-carboxyethyl)phosphine hydrochloride] and 10 mM iodoacetamide at room temperature in the dark while being mixed at 1,200 rpm in an Eppendorf thermomixer. Proteins were then digested by the addition of 0.1 μg Lys-C (Fujifilm Wako Pure Chemical Corporation; 125-05061) and 0.8 μg trypsin (Thermo Scientific; 90057) with shaking at 37°C overnight. The digestions were quenched via addition of formic acid to a final concentration of 5% by volume. Each sample was desalted via C_18_ tips (Thermo Scientific, 87784) and then resuspended in 15 μl of 5% formic acid before analysis by liquid chromatography-tandem mass spectrometry (LC-MS/MS).

### LC-MS acquisition and analysis.

Peptide samples were separated on a 75-μm (inside diameter), 25-cm C_18_ column packed with 1.9-μm C_18_ particles (Dr. Maisch GmbH) using a 140-min gradient of increasing acetonitrile and eluted directly into a Thermo Orbitrap Fusion Lumos instrument, where MS/MS spectra were acquired by data-dependent acquisition (DDA). Data analysis was performed using the ProLuCID (48) and DTASelect2 (49, 50) algorithms as implemented in the Integrated Proteomics Pipeline (IP2; Integrated Proteomics Applications, Inc., San Diego, CA). Protein and peptide identifications were filtered using DTASelect and required a minimum of two unique peptides per protein and a peptide-level false-positive rate of less than 1% as estimated by a decoy database strategy.

### Data availability.

All mass spectrometry data are accessible at the MassIVE data set repository under the identifier MSV000087615 (https://massive.ucsd.edu/ProteoSAFe/static/massive.jsp) (51).
