# Supplementary figures and images for: A Novel Toxoplasma Inner Membrane Complex Suture-Associated Protein Regulates Suture Protein Targeting and Colocalizes with Membrane Trafficking Machinery
Source: mBio. 2021 Oct 12;12(5):e02455-21. doi: 10.1128/mBio.02455-21 (PMC8510555; doi:10.1128/mBio.02455-21)

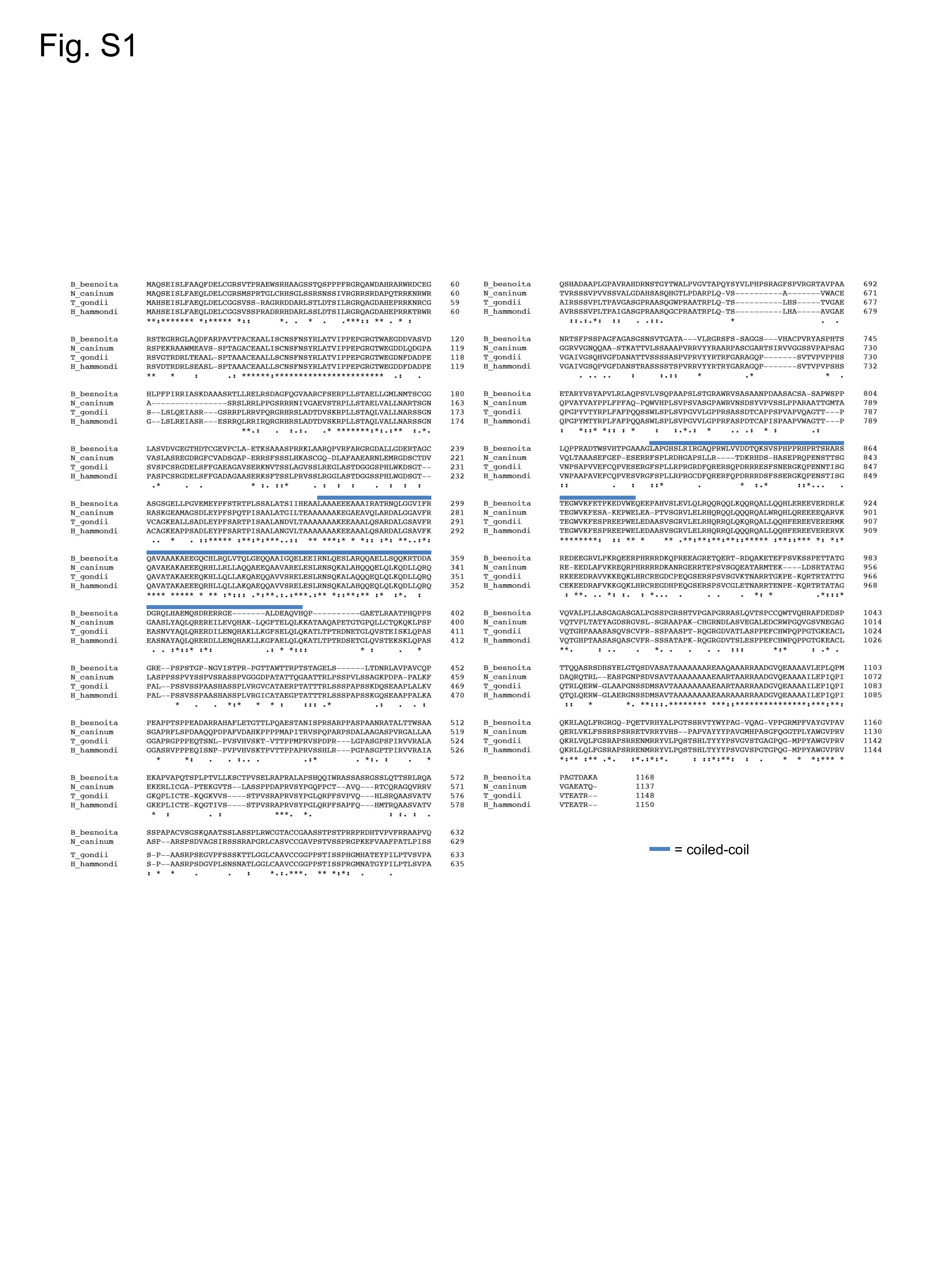

Supplement: FIG S1 [file mbio.02455-21-sf001.tif]

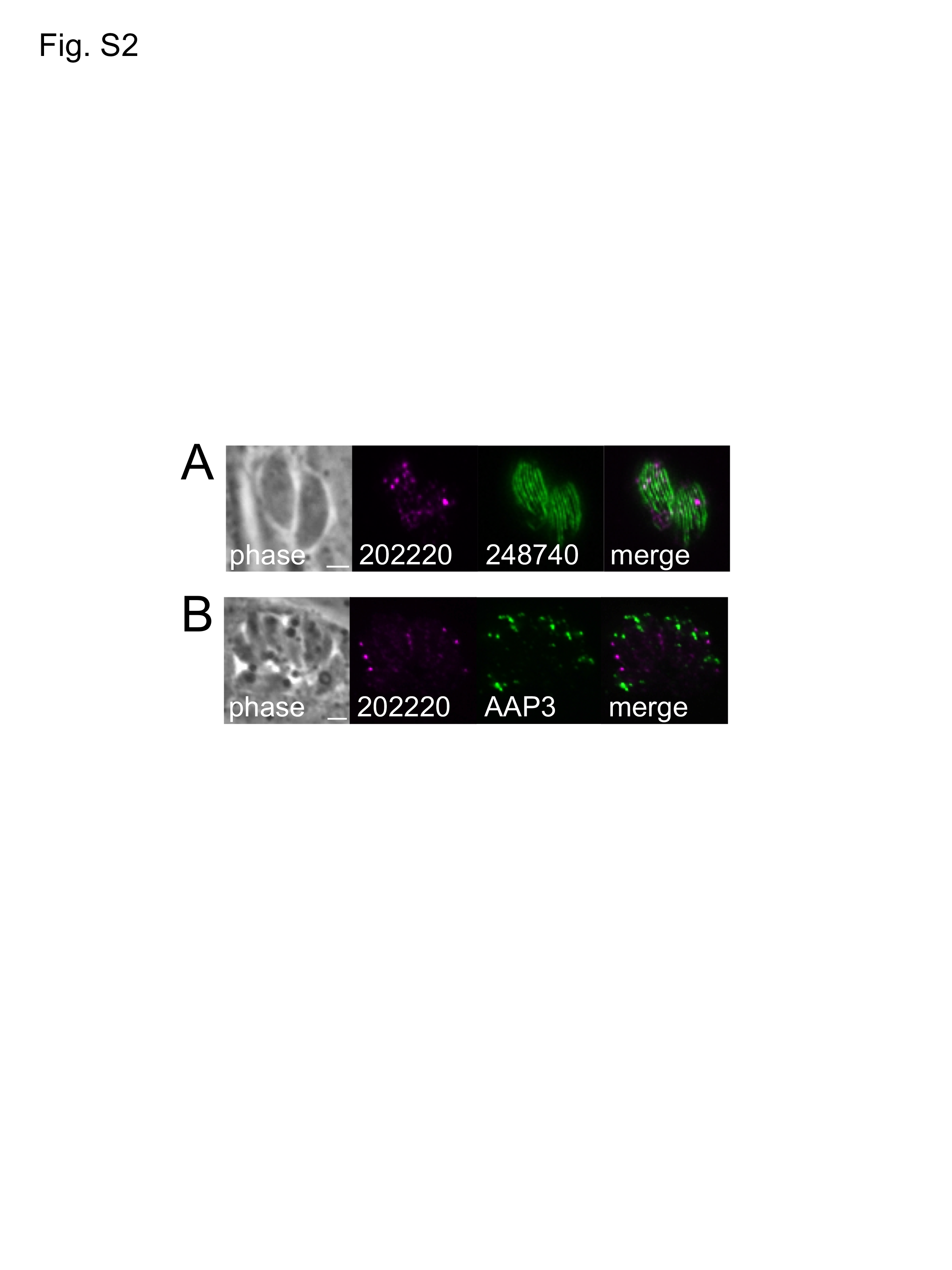

Supplement: FIG S2 [file mbio.02455-21-sf002.tif]

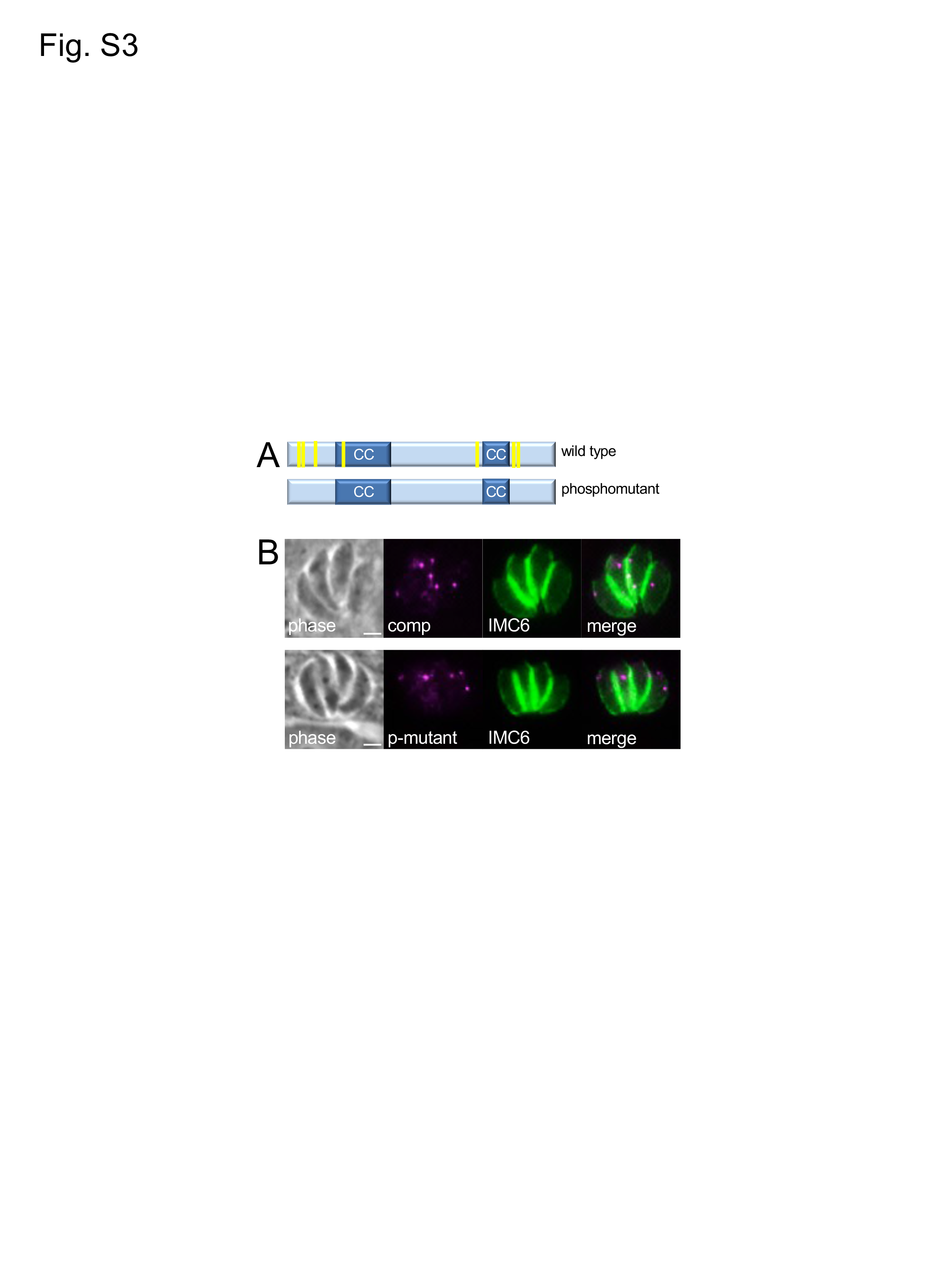

Supplement: FIG S3 [file mbio.02455-21-sf003.tif]

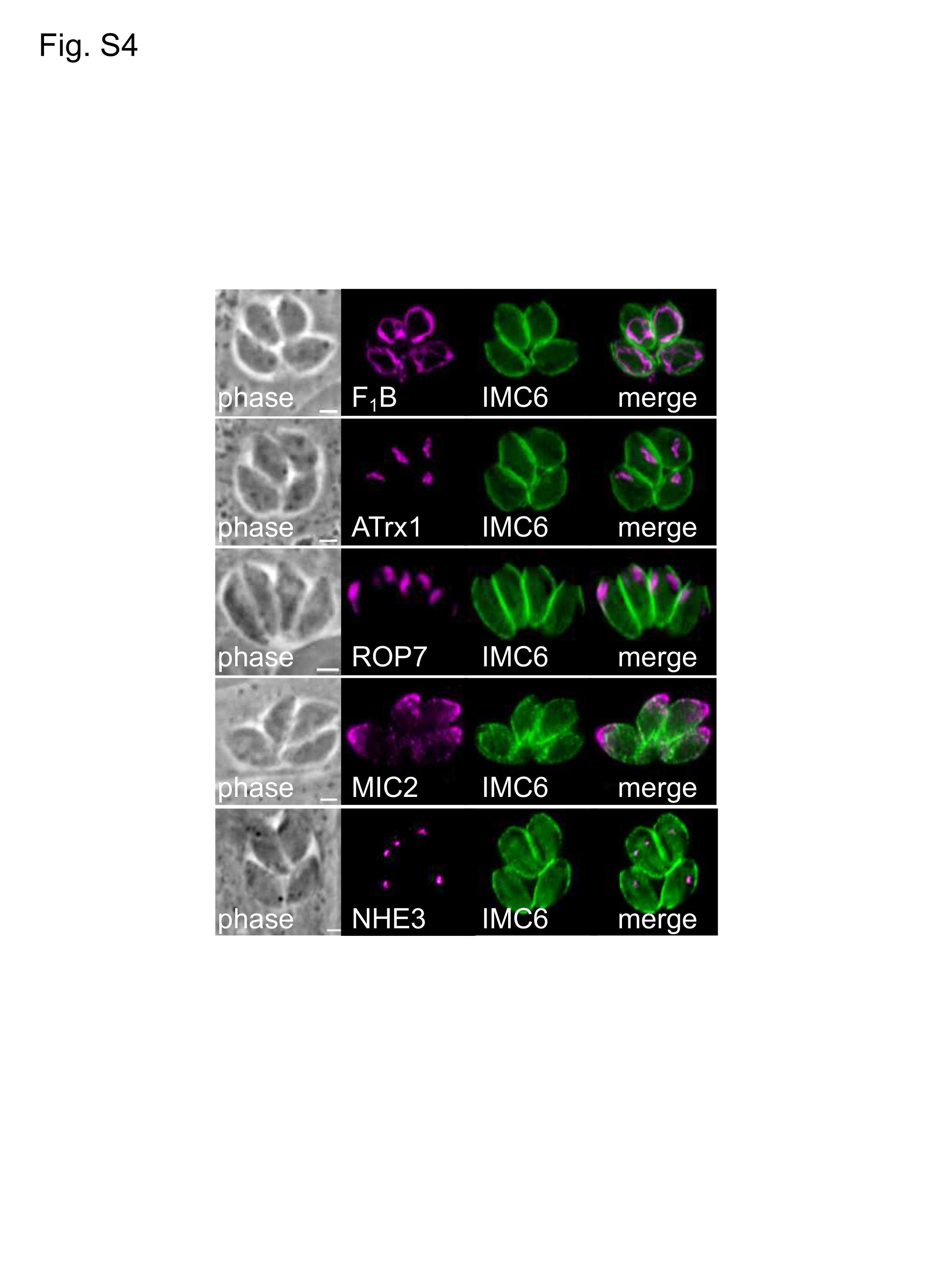

Supplement: FIG S4 [file mbio.02455-21-sf004.tif]

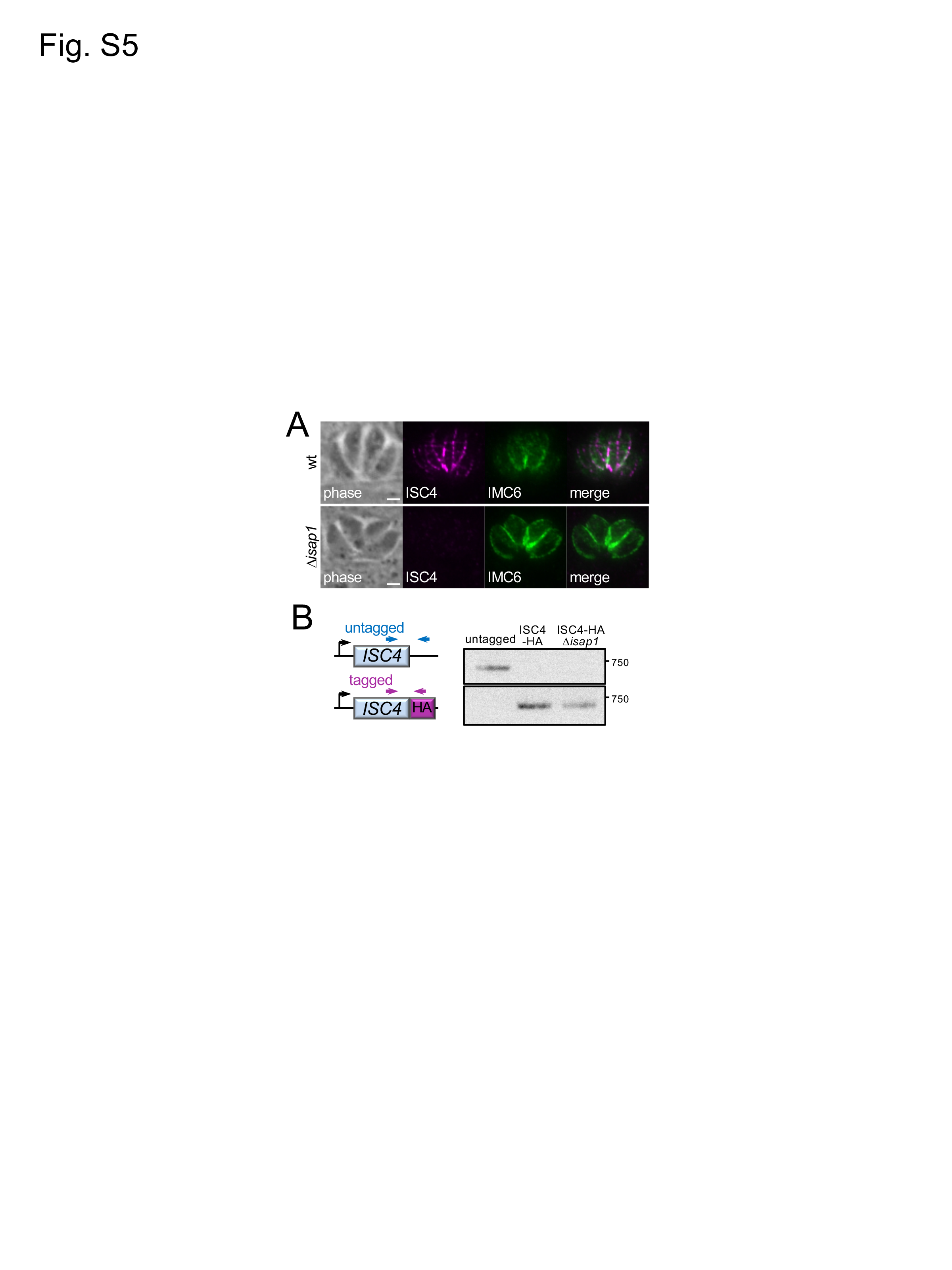

Supplement: FIG S5 [file mbio.02455-21-sf005.tif]

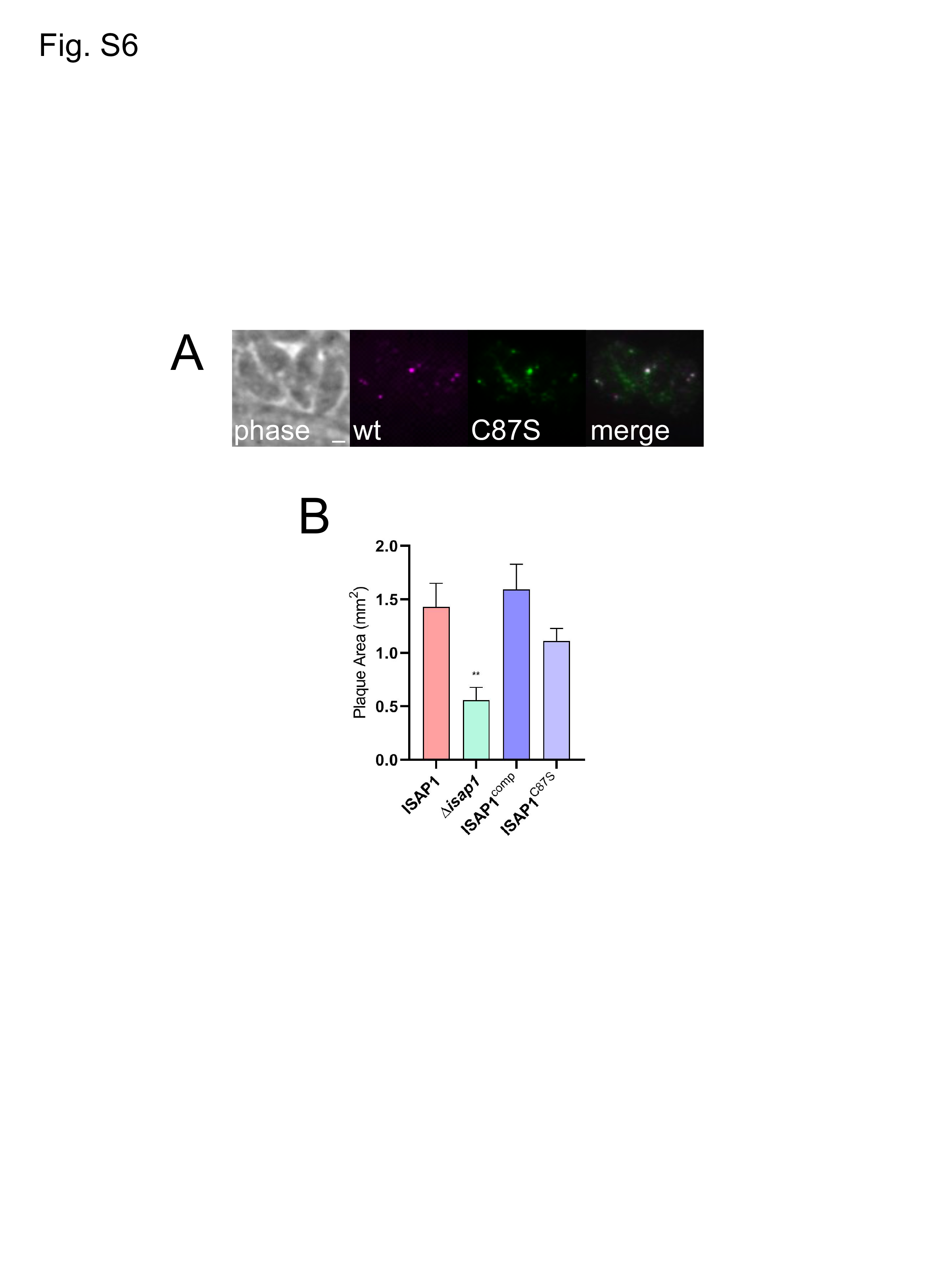

Supplement: FIG S6 [file mbio.02455-21-sf006.tif]
