# Supplementary material for: A Novel Toxoplasma Inner Membrane Complex Suture-Associated Protein Regulates Suture Protein Targeting and Colocalizes with Membrane Trafficking Machinery
Source: mBio. 2021 Oct 12;12(5):e02455-21. doi: 10.1128/mBio.02455-21 (PMC8510555; doi:10.1128/mBio.02455-21)
Supplement: TABLE S1 [file mbio.02455-21-st001.pdf]

Name	Description	Sequence
P1	202220 universal homology	TGCATGGGGAGTGCCTCGTGTGGTTACCGAAGCAACGCGGGGAAGTGGAGGACGGG
P2	202220 universal homology	GTTATGTAGTCTTCCAACACATAACTAGTTGGAGAGTGCGACGGCCAGTGAATTGTA
P3	202220 gRNA tag fwd	AAGTTGATTGGCTGACTTGAAGCGAG
P4	202220 gRNA tag rev	AAAACTCGCTTCAAGTCAGCCAATCA
P5	202220 HDRko fwd	TTGCCCTTTCAGGACTCGCGTTTTTTTGCAGAGTGCAAACCACTCCATGGAACCTGA
P6	202220 HDRko rev	CTGGCGGTGTCTACGCAAAAAGCATTTATCTCACGACTGCCTGCAAGTGCATAGAAG
P7	202220 gRNA 1 ko fwd	AAGTTGGAGGGTACCCCAAAGAGGGG
P8	202220 gRNA 1 ko rev	AAAACCCCTCTTTGGGGTACCCTCCA
P9	202220 gRNA 2 ko fwd	AAGTTGACCTACTACTACCCTTCAGG
P10	202220 gRNA 2 ko rev	AAAACCTGAAGGGTAGTAGTAGGTCA
P11	202220 gene check fwd	CGCGACAGATTGACTGAAGCT
P12	202220 gene check rev	CTGAAGCAGGTGCTTCTGTTC
P13	202220 5' UTR check fwd	GAGAGCATCGTGATTCGCTTG
P14	NcGra7 HPT rev	CAGGTCTCGCAAGCAGTTC
P15	202220 comp1 fwd	GGATCCATGGCACATTCTGAGATATCCTTATTC
P16	202220 comp1 rev	GAGAACATCATTGGCAAGAGCG
P17	202220 comp2 fwd	CGACAGATTGACTGAAGCTGC
P18	202220 comp2 rev	GCGGCCGCCCGCGTTGCTTCGGTAACC
P19	202220 deletion (268-384) fwd	AAAGCGACTTTGACCCCG
P20	202220 deletion (268-384) rev	TGCAGCCGCAGTGAGAAC
P21	202220 deletion (867-923) fwd	CACCGCTGCCGTGAGGGG
P22	202220 deletion (867-923) rev	ATCTTCCAGCTCCCACGGTTCC
P23	202220 deletion (1041-1148)	GCGGCCGCATACCCGTAC
P24	202220 deletion (1041-1148)	CGAGTTGCCAGGTGCAGC
P25	202220 deletion (2) rev	CATGGATCTTGCGTCGGC
P26	202220 deletion (2-250) fwd	CGCACTCCAATTTCGGCC
P27	202220 deletion (2-392) fwd	GACAACGAGACAGGTCTC
P28	202220 deletion (811-1148)	CAGCAACGGGGAAAAGCCC
P29	202220 deletion (930-1148)	CCCCTCACGGCAGCGGTG
P30	202220 deletion (2-121) fwd	TTGCAGGAAATCGCTTCC
P31	202220 deletion (2-180) fwd	GGTGACGAGCTAAGTTTCTTTG
P32	UPRT - 202220 promoter fwd	GTTCGACATCGCATATGCATACGGCGGCGTCTTTCTCTTTTC
P33	202220 genomic - SM MYC	GATATCAGTTTTTGCTCCATCCGCGTTGCTTCGGTAAC
P34	SM MYC fwd	ATGGAGCAAAAACTGATATCG
P35	3 UPRT flank rev	ATGCATATGCGATGTCGAAC
P36	227800 universal homology	GCCCGTCGCGGGTTTACGCGGTCTTGCTGCAGCTTCTCTCGGAAGTGGAGGACGGGA
P37	227800 universal homology	ACTCTCTACGCACAAATGCCATTGCCTTCAGAACACTGCGACGGCCAGTGAATTGTA
P38	227800 gRNA tag fwd	AAGTTGAAGGCTGGCGTGTATAGTGG
P39	227800 gRNA tag rev	AAAACCACTATACACGCCAGCCTTCA
P40	297520 universal homology	AGTGCCTTCTTCGCTTCCCGGCGCCAACTCTCCCTTCCGAGGAAGTGGAGGACGGGA
P41	297520 universal homology	GGAGACAGGACCTCTTGAGACAGAAGAGAACGCAGAGGCGACGGCCAGTGAATTG
P42	297520 gRNA tag fwd	AAGTTGAGAGAGTCGCAAAAACTCGG
P43	297520 gRNA tag rev	AAAACCGAGTTTTTGCGACTCTCTCA
P44	272600 universal homology	GGCCACACCCCAACCGGCGCCTTTGCCGCTCTCTTCGAGCGGAAGTGGAGGACGGG
P45	272600 universal homology	GACCTGCTCGCTCCCAAGCAACACAGATTCATATACATCGACGGCCAGTGAATTGTA
P46	272600 gRNA tag fwd	AAGTTGAACGGGCAACATATCTATCCG
P47	272600 gRNA tag rev	AAAACGGATAGATATGTTGCCCGTTCA
P48	270690 fwd univ homology	AGGAAGTTCCGGTCGGCTTCCGTCACCGTTGAATGGGGCTGGAAGTGGAGGACGGG
P49	270690 rev univ homology	CACACGACCTTTTGGTCTCCTCTACAGTGTCTGACTTGCGACGGCCAGTGAATTGTA
P50	270690 gRNA tag fwd	AAGTTGAAGGGACAACTGTTCTGCGG
P51	270690 gRNA tag rev	AAAACCGCAGAACAGTTGTCCCTTCA
P52	ISC6 universal homology fwd	TTGTTCTGTTTTTTTTTCGCAGGCGAAAGGAGGCGAGTGCGGAAGTGGAGGACGGGA
P53	ISC6 universal homology rev	GCAGGAAAGAGAGAGCGAGGAAAGGAAGAACAACATGCCGACGGCCAGTGAATTG
P54	ISC6 gRNA tag fwd	AAGTTACGAGTGGACCACTGCGCTGG
P55	ISC6 gRNA tag rev	AAAACCAGCGCAGTGGTCCACTCGTA
P56	ISC3 universal homology fwd	CCTCAGGGAGTTGCTTCTCGACGCCCAGCAAGAAATGATGGGAAGTGGAGGACGGG
P57	ISC3 universal homology rev	CGCTGACAGAGTCACTCCTCAGCAGTCAAGTGAATACGCGACGGCCAGTGAATTGT
P58	ISC3 gRNA tag fwd	AAGTTGACAGAGCGTCCACGAAAAAG
P59	ISC3 gRNA tag rev	AAAACTTTTTCGTGGACGCTCTGTCA
P60	ISC4 universal homology fwd	AGTGCAGTCTTCCCCAGCAACCTGGATCACTATGTGCGTGGAAGTGGAGGACGGGA
P61	ISC4 universal homology rev	ATGACTCTTCACATGCTTCGCACACCCTCATGCCTTCTCGACGGCCAGTGAATTGTA
P62	ISC4 gRNA tag fwd	AAGTTACCGGGGTCAGATCTGGCAAG
P63	ISC4 gRNA tag rev	AAAACTTGCCAGATCTGACCCCGGTA
P64	ISC4 check fwd	CTGCACCTTCCAGTTCTTG
P65	ISC4 3'UTR check rev	GAAGAGAGGAACCAAAGGG
P66	HA tag check rev	CATCATAGGGATAGCCAGCG
P67	AAP3 universal homology fwd	TACAGAAGAAATCGCAGCTCTTGTTGAAGTTGATTTGTCGGGAAGTGGAGGACGGG
P68	AAP3 universal homology rev	GAAATGTGTGTTGGACACGAGATGTGTTCCTCGGACTCCGACGGCCAGTGAATTGTA
P69	AAP3 gRNA tag fwd	AAGTTACGCCTGCCTTCACATGTTCG
P70	AAP3 gRNA tag rev	AAAACGAACATGTGAAGGCAGGCGTA

Table S1. Oligonucleotide primers used in this study. All primer sequences are shown in the 5' to 3' orientation.
